# Effect of three substituted dihydroimidazole corrosion inhibitors on carbon steel surfaces: experimental and theoretical studies of inhibition and adsorption performance

**DOI:** 10.1039/d5ra03853g

**Published:** 2025-10-21

**Authors:** A. Marzaq, M. El Faydy, Daniil R. Bazanov, Natalia A. Lozinskaya, M. Maatallah, G. Kaichouh, M. Allali, L. Bazzi, A. Dafali, A. Zarrouk

**Affiliations:** a Laboratory of Molecular Spectroscopy Modelling, Materials, Nanomaterials, Water and Environment, CERNE2D, Faculty of Sciences, Mohammed V University in Rabat Morocco azarrouk@gmail.com +00212665201397; b Laboratory of Applied Chemistry and Environment (LCAE), Faculty of Sciences, Mohammed First University Oujda 600 Morocco; c Department of Chemistry, Lomonosov Moscow State University 119991 Moscow Russia; d Laboratory of Molecular Chemistry, Faculty of Sciences Semlalia, Cadi Ayyad University PO Box 2390 Marrakech Morocco; e Institute of Nursing Professions and Health Techniques Fez, EL Ghassani Hospital Fez 30000 Morocco; f Laboratoire de Génie Industriel, de L’Énergétique et de L'Environnement (LGI2E) SupMTI Rabat 10000 Morocco; g Laboratory of Materials, Nanotechnology and Environment, Faculty of Sciences, Mohammed V University in Rabat Av. Ibn Battouta, P.O. Box. 1014 Agdal-Rabat Morocco

## Abstract

Corrosion resistance and adsorption attributes of three synthesized dihydroimidazoles, namely (4R,5S)-2,4,5-tris(3,4,5-trimethoxyphenyl)-4,5-dihydro-1*H*-imidazole (TMPI), (4R,5S)-2,4,5-tris(4-(methylthio)phenyl)-4,5-dihydro-1*H*-imidazole (MSPI), and (4S,5R)-2,4,5-tri(thiophen-2-yl)-4,5-dihydro-1*H*-imidazole (TTPI), were scrutinized for carbon steel in 1 M HCl employing a combination of analytical techniques, including electrochemical and instrumental, alongside quantum computational methods. Electrochemical experiments revealed that the effectiveness of corrosion inhibition is influenced by the substituted dihydroimidazoles' concentration and structure. The inhibition efficiency reached remarkable levels: 98.53% for TTPI, 98.59% for TMPI, and 99.12% for MSPI at 0.001 M. The Tafel polarization method indicated that these synthesized dihydroimidazoles function as mixed-type inhibitors. The adsorption process of synthesized dihydroimidazoles adheres to the Langmuir adsorption isotherm. Various analytical techniques, including scanning electron microscopy (SEM), energy-dispersive X-ray spectroscopy (EDS), contact angle, atomic force microscopy (AFM), and X-ray diffraction analysis (XRD), confirmed that substituted dihydroimidazoles form a protective layer that inhibits carbon steel dissolution. Furthermore, molecular dynamics (MD) was utilized to investigate the adsorption behavior of substituted dihydroimidazoles on the Fe (110) surface, determining the entanglement order of their interaction. The radial distribution function (RDF) revealed that corrosion inhibitors employ a complex adsorption mechanism on metal surfaces, primarily driven by chemical processes and supplemented by physical adsorption.

## Abstract 2

MSPI achieves exceptional inhibitory efficacy of 99.12% in 1 M HCl. The three dihydroimidazoles act as mixed inhibitors with Langmuir adsorption. MD simulations confirm stable and parallel adsorption on the Fe(110) surface.

## Graphical abstract

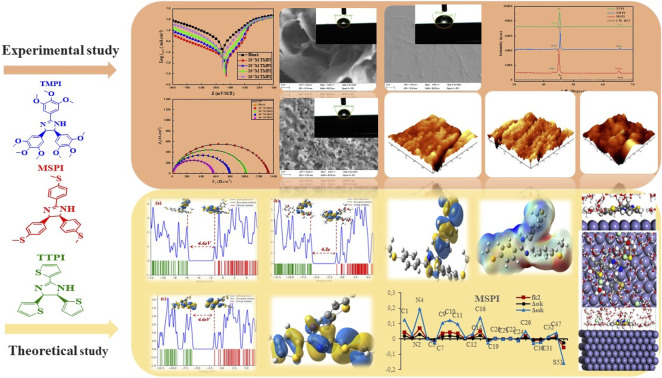

## 1. Introduction

The deterioration of carbon steel due to corrosion has become a significant issue, attracting the attention of many researchers in recent years. This is largely due to the considerable environmental harm it causes, the decline of industrial equipment, and the substantial economic losses it incurs. Global estimates suggest that corrosion-related economic impacts range from 3% to 6% of the world's gross domestic product annually.^1^ In numerous industrial sectors, carbon steel is preferred for its exceptional mechanical properties, widespread availability, and versatility in equipment production.^2^ Hydrochloric acid (HCl) is significant in various treatments such as hydrometallurgical, steel pickling, and mineral and chemical processes.^3^ When steel is exposed to these acidic conditions, it undergoes substantial corrosion-induced degradation, which negatively impacts the performance and lifespan of industrial equipment. This situation highlights the pressing need to develop effective corrosion mitigation strategies.^4,5^ Among the various corrosion prevention methods, corrosion inhibitors have gained prominence.^6–8^ This is attributed to their numerous advantages, including low consumption rates, established technological frameworks, simple application procedures, and cost-effectiveness.^9,10^ Corrosion inhibitors are generally divided into two main categories based on their chemical makeup: traditional inorganic inhibitors and synthetic organic inhibitors. Key commercial inorganic inhibitors, including chromates, phosphates, nitrates, and molybdates, are suitable for use in near-neutral environments.^11,12^ These inhibitors are known for their long-lasting corrosion prevention at high temperatures and are economically viable. However, their high toxicity to ecosystems and lack of biodegradability have led to their prohibition.^13^ Consequently, there has been a growing emphasis on the use of organic inhibitors in research. Organic corrosion inhibitors, particularly N-heterocyclic compounds, have become widely adopted.^14,15^ These compounds are well-suited for acidic conditions due to their remarkable stability and durability. They function by forming a protective layer through the movement of the π electron cloud towards N atoms, creating a coordinating effect.^16^ Their versatile nature allows them to be tailored to various corrosion environments, enhancing adsorption and mitigating issues related to surface roughness, overuse, or environmental factors that might impact the performance of traditional inhibitors.^17–20^ The effectiveness of these molecules is determined by their functional groups.

Imidazole analogs, a crucial category of N-heterocyclic compounds, are widely utilized in pharmaceuticals and environmentally friendly agricultural chemicals.^21–23^ The imidazole scaffold structure is present in many natural compounds, such as histamine, histidine, purine, and nucleic acids, which are known for their low environmental toxicity.^24^ Furthermore, research has shown that imidazoles are effective in preventing metal corrosion.^25–27^ Jrajri *et al.* explored the use of two imidazoles, abbreviated as TOIE and TFMI, as corrosion inhibitors for carbon steel in molar HCl through various experimental and empirical methods. Their findings indicated that imidazoles function as strong inhibitors due to the high electron density in the nitrogen atoms, achieving corrosion resistance of 94.9% for TOIE and 89.2% for TFMI at 0.001 M under 303 K.^28^ In contrast, Prashanth *et al.* employed both practical and computational methods to examine the substituted effect of OH, NH_2_, and OCH_3_ groups in three imidazoles abbreviated (IM1), (IM2), and (IM3) in acidizing mild steel in 1/2 M HCl. They found that the greater inhibition efficiency of IM1 with a more electron-donating group (OH) has more absorption capability on the mild steel surface compared to IM2 with OCH_3_ and IM3 with NH_2_ groups.^29^ Another study carried out by El-Haddad *et al.* considered the effectiveness of imidazole and methylimidazole for pure aluminum corrosion in 0.5 M HCl using various techniques. They deduced that methylimidazole exhibits a performance of 76% at 18 × 10^−5^ M at 30 °C.^30^ Substituted imidazoles are recommended for creating novel corrosion inhibitors owing to the many synthesis techniques accessible and their unique structural interactions, which allow vast applications in applied chemistry.

The main novelty of our study lies in synthesizing three new substituted dihydroimidazoles: (4R,5S)-2,4,5-tris(3,4,5-trimethoxyphenyl)-4,5-dihydro-1*H*-imidazole (TMPI), (4R,5S)-2,4,5-tris(4-(methylthio)phenyl)-4,5-dihydro-1*H*-imidazole (MSPI), and (4S,5R)-2,4,5-tri(thiophen-2-yl)-4,5-dihydro-1*H*-imidazole (TTPI), along with their characterization using spectroscopic methods. They were synthesized from substituted aldehydes and ammonia in two simple steps under mild conditions. The effectiveness of these dihydroimidazoles as corrosion inhibitors for carbon steel in 1 M HCl was initially evaluated through electrochemical tests. Additionally, surface morphology analyses with and without the inhibitors were performed using SEM-EDS, XRD, AFM, and contact angle measurements. Structure–activity relationships were explored through MD, DFT, and MC studies. Although many studies have focused on dihydroimidazole-based inhibitors, their efficiency in preventing steel corrosion has been limited.^25–29^ In contrast, our derivatives achieved inhibition efficiencies of up to 99%, exceeding most of those cited in previous studies under similar conditions. This significant improvement results from our innovative molecular design that enhances active sites, aromaticity, and electron density, thereby increasing adsorption capacity and surface coverage on the carbon steel substrate.

## 2. Experimental

### 2.1. Material and corrosive solution

Carbon steel strips with an area of 1 cm^2^ were employed for electrochemical analysis. The chemical composition of steel is as follows: 0.370% carbon (C), 0.230% silicon (Si), 0.680% manganese (Mn), 0.016% sulphur (S), 0.077% chromium (Cr), 0.011% titanium (Ti), 0.059% nickel (Ni), 0.009% cobalt (Co), 0.160% copper (Cu), with the remainder being iron (Fe). The samples underwent polishing with emery sheets of grades 180, 400, 1000, and 1200. Furthermore, they were thoroughly rinsed in distilled water multiple times and stored in a desiccator. Meanwhile, the corrosive solution, 1 M HCl, was prepared by diluting reagent-grade hydrochloric acid (37% Merck) with distilled water.

### 2.2. Dihydroimidazoles synthesis

Dihydroimidazoles were synthesized from the corresponding aldehydes *via* a one-step procedure, as described previously.^31^ Briefly, 5 g of the appropriate aldehyde (3,4,5-trimethoxybenzaldehyde, 4-((methylthio)benzaldehyde, or thiophene-2-carbaldehyde) was added to a solution of ammonia in tetrahydrofuran (for the trimethoxy and methylthio derivatives) or to 25% aqueous ammonia (for the thiophene derivative). The reaction mixtures were stirred at room temperature for 48 h. For the trimethoxy and methylthio derivatives, the solvent was removed under reduced pressure; for the thiophene derivative, the intermediate was isolated by filtration using a Schott filter. The crude diazapentaene intermediates were dissolved in THF (50 mL), and potassium *tert*-butoxide (2–3 g) was added. Upon disappearance of the intense blue or violet color, the reaction mixtures were diluted with water to a total volume of 500 mL and stirred for 24 h in open beakers at ambient temperature. The resulting precipitates were collected by filtration through a Schott filter and washed with a small amount of petroleum ether or cold diethyl ether to remove residual aldehyde. Structures were confirmed by NMR spectroscopy, and their spectra are merged in the SI file (Table 1 and Fig. S1–S9). The products were used in corrosion testing without further purification.

**Table 1:** Nomenclature, acronyms, molecular structure, and NMR data of the dihydroimidazoles

Name (acronym)	Molecular structure	Yield	^1^H NMR (400 MHz, CDCl_3_) *δ*_ppm_	^13^C NMR (101 MHz, CDCl_3_) *δ*_ppm_
(4R,5S)-2,4,5-tris(3.4.5-trimethoxyphenyl)-4,5-dihydro-1*H*-imidazole (TMPI)	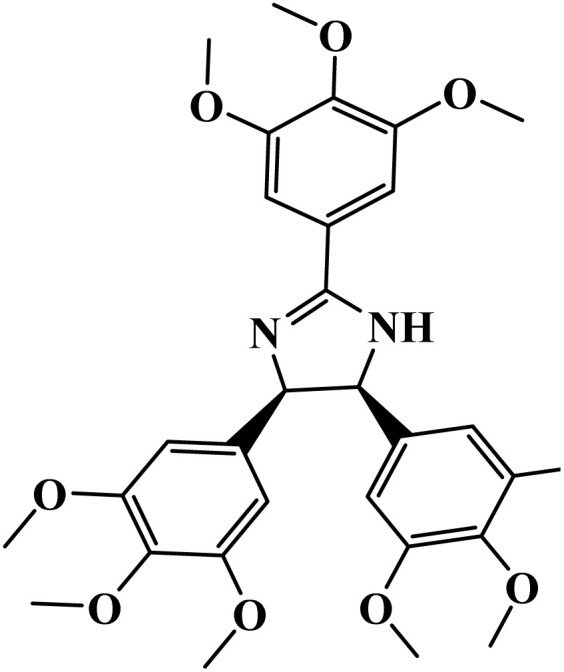	88%	7.21 (s, 2H), 6.12 (s, 4H), 5.54 (s, 1H), 5.43 (s, 1H), 3.91 (s, 9H), 3.69 (s, 6H), 3.63 (s, 12H)	164.6, 153.3, 152.6, 134.5, 125.3, 105.2, 104.5, 75.0, 66.6, 61.0, 60.8, 56.4, 56.0
(4R,5S)-2,4,5-tris(4-(methylthio)phenyl)-4,5-dihydro-1*H*-imidazole (MSPI)	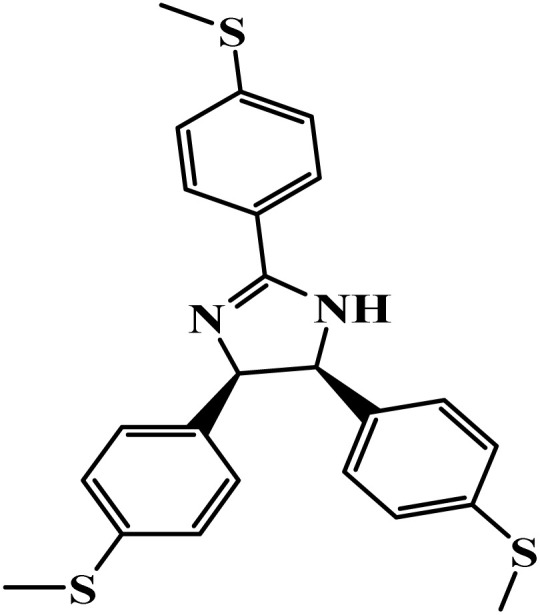	85%	7.82 (d, *J* = 7.9 Hz, 2H), 7.29–7.24 (m, 2H), 6.92 (d, *J* = 7.9 Hz, 4H), 6.82 (d, *J* = 8.5 Hz, 4H), 5.30 (s, 2H), 2.52 (s, 3H), 2.35 (s, 6H)	163.8, 142.5, 136.3, 135.5, 127.6, 127.2, 125.8, 125.2, 15.6, 14.7
(4S,5R)-2.4.5-tri(thiophen-2-yl)-4,5-dihydro-1*H*-imidazole (TTPI)	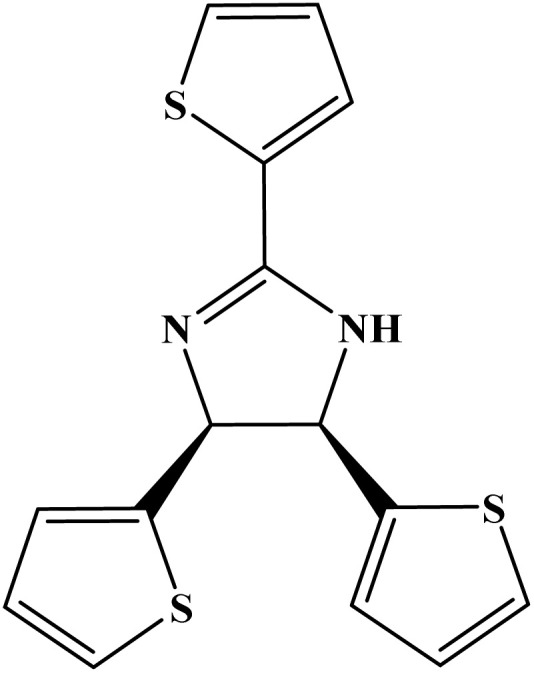	85%	7.52 (d, *J* = 3.7 Hz, 1H), 7.50 (d, *J* = 5.0 Hz, 1H), 7.12 (t, *J* = 4.4 Hz, 1H), 7.07 (d, *J* = 5.0 Hz, 2H), 6.81 (t, *J* = 4.3 Hz, 2H), 6.75 (d, *J* = 3.5 Hz, 2H), 5.60 (s, 2H)	159.0, 141.6, 132.5, 129.2, 127.9, 127.2, 125.9, 125.0, 124.4, 77.0, 76.6, 76.3, 66.3

Due to their heterocyclic ring structure containing nitrogen atoms that readily protonate in hydrochloric acid, the studied dihydroimidazoles exhibit high solubility in HCl. Consequently, the dihydroimidazoles were directly dissolved in 1 M HCl to prepare stock solutions at a concentration of 10^−3^ M. These stock solutions were then serially diluted with 1 M HCl to achieve the desired concentration range, traveling from 10^−4^ M to 10^−6^ M.

### 2.3. Electrochemical analysis

An electrochemical examination was achieved utilizing a three-electrode structure connected to a potentiostat (PGZ 100). The system comprised a carbon steel electrode (1 cm^2^) as the working electrode, a platinum electrode as the auxiliary electrode, and a saturated calomel electrode (SCE) as the reference electrode. The electrolyte solution was 1 M HCl dissolved in deionized water. To earn a stable open circuit potential (OCP), the working electrode was submerged in each corrosive solution for 1800 seconds prior to each test. PDP measurements were recorded between −800 and −100 mV/OCP at a sweep rate of 0.5 mVs^−1^. EIS was conducted over a frequency range from 10 mHz to 100 kHz with a 10 mV perturbation. EIS data analysis was carried out utilizing ZView software. The corrosion potential (*E*_corr_), corrosion current density (*i*_corr_), and various resistances were derived from the PDP curve utilizing Origin software (Origin Lab). The inhibition efficiencies obtained from PDP (*η*_p_ (%)) and EIS (*η*_imp_ (%)) measurements are determined as below:^32^1
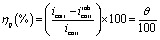
where *i*_corr_ and *i*^inh^_corr_ (μA cm^−2^) denote the corrosion current densities under dihydroimidazole's influence and in its absence, respectively.2
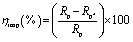
where *R*_p_ and 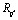 (Ω cm^2^) denote polarization resistances under dihydroimidazole's influence and in its absence, respectively.

### 2.4. Surface analysis

After 6 hours of immersion, the carbon steel specimens treated with the various dihydroimidazole compounds were carefully dried and subjected to surface characterization. Scanning electron microscopy coupled with energy-dispersive X-ray spectroscopy (SEM-EDS, JEOL JSM-IT-100 Scientific) and atomic force microscopy (AFM, Bruker Dimension Icon) were employed to examine the surface morphology and elemental distribution. Crystalline structure analysis was performed using an Empyrean X-ray diffractometer. Additionally, the surface hydrophobicity was evaluated *via* contact angle measurements using a Dataphysics OCA 50.

We would like to emphasize that the electrochemical and surface analysis results of the uninhibited solution in this study are identical to those reported in our previous publication. This is because we conducted the experiments under identical conditions and using the same equipment.^33^

### 2.5. Quantum chemical calculations

The results of our experimental studies indicate that the TMPI, MSPI, and TTPI have notable effectiveness as corrosion inhibitors, suggesting that they have a strong interaction with the carbon steel surface. However, to truly understand the phenomena at the molecular scale and elucidate the adsorption processes of these molecules on the metallic iron surface, a detailed analysis of their electronic and structural properties is essential. Thus, the present theoretical research is based on the theory of the density functional (DFT) in order to examine the characteristics of the three dihydroimidazoles TMPI, MSPI, and TTPI. The aim is to validate and interpret the experimental results by establishing a correlation between the molecular structure of the compounds and their inhibition efficiency. We have sought to determine the most stable configurations of these dihydroimidazoles, both in the neutral and protonated forms. For this, their geometries have been optimized using the hybrid functional B3LYP and the 6-311G(d.p) basic set,^34^ with the Gaussian09 software.^35^ The absence of imaginary frequencies has been confirmed by a frequency calculation, which guarantees the stability of the structures obtained. The protonated forms were also examined in order to evaluate the impact of the charge and the geometry on their adsorption and their interaction with the Fe(110) surface.

We then explored the key electronic properties of TMPI, MSPI, and TTPI, which play an essential role in their interaction with metal surfaces. Based on the results of quantum calculations, we analyzed several chemical reactivity parameters based on the energies of the frontier molecular orbitals, the highest occupied and lowest unoccupied molecular orbitals (HOMO and LUMO, respectively). These parameters have provided us with a detailed vision of the electronic properties and the reactivity of the molecules studied. The main parameters, calculated following the equations described in the literature,^36^ are the energy gap (Δ*E* = *E*_LUMO_ – *E*_HOMO_), the ionization potential (*I*), and the electron affinity (*A*), which are indicators of the tendency of molecules to lose or gain electrons. The global hardness (*η*) and the softness (*σ*) measure the resistance of the molecule to changing its electronic distribution. Absolute electronegativity (*χ*) and the fraction of electrons transferred (Δ*N*) are essential parameters to estimate the capacity of electron transfer between the molecule and the carbon steel surface. The global electrophilicity (*ω*) evaluates the tendency of the molecule to receive electrons. And also, the electron back-donation energy (Δ*E*_back-donation_).

We also evaluated the molecular electrostatic potential (MEP) to identify the region most likely to react within molecules and deepen our understanding of their function as electron donors or acceptors. To evaluate the possible interactions with the metal, we examined the local reactive sites for each k atom using dual Fukui descriptor *f*_k_^2^,^37^ dual local softness (Δ*σ*_k_), and dual local philicity (Δ*ω*_k_).^38^

### 2.6. *Ab initio* DFT simulations

To better understand the adsorption mechanism, we studied the interactions of our organic compounds on the Fe(110) surface using the spin-polarized density functional theory (DFT), *via* the CASTEP program (Cambridge sequential total energy program).^39,40^ The exchange–correlation energy has been described by the generalized gradient approximation (GGA) parameterized with the PBE functional, while the dispersion effects, such as van der Waals interactions, have been taken into account thanks to the DFT-D method from Grimme. The geometric optimization was carried out using the BFGS algorithm, applying strict convergence criteria for displacement. Force and total energy (with tolerances of 1.0 × 10^−5^ eV per atom and 0.03 eV Å^−1^). The core electrons have been replaced by Vanderbilt ultrasoft pseudopotentials, thus simplifying the calculations while maintaining accuracy. For adsorption systems, a model of Fe-inhibitor was constructed using an Fe(110) surface comprising a 6 × 6. These choices make it possible to ensure an accurate description of the electronic and structural properties of the system.

### 2.7. Monte Carlo and molecular dynamics simulation details

MC and MD simulations of the adsorption of the three dihydroimidazoles (TMPI, MSPI, and TTPI) on the surface of carbon steel were carried out using the Materials Studio^41^ software. To model this interaction, we used the COMPASS III force field (Condensed phase Optimized Molecular Potentials for Atomistic Simulation Studies),^42^ which is particularly suitable for condensed phase studies. The carbon steel surface was represented by the crystalline plane (110), and a supercell of dimensions 10 × 10 was created with a vacuum space of 30 Å with periodic boundary conditions to avoid interactions between the periodic images. In order to determine the most stable and spontaneous configuration of the inhibitory molecules on the surface of Fe(110), we took into account the effects of the solvent by integrating 250 water molecules into the system. To imitate the real conditions of a corrosive medium, the simulation cell was completed with 5 hydronium ions (H_3_O^+^) and 5 chloride ions (Cl^−^).

MD calculations were performed using the NVT set. A time step of 1 fs was chosen, with a total simulation duration of 1000 ps. The system temperature was maintained at 303 K and regulated using Andersen's thermostat, which allows efficient temperature control while preserving the dynamic properties of the system. The interaction energy (*E*_Interaction_) between the molecules and the Fe(110) surface was calculated to evaluate the stability of the adsorption according to the following equation:^43^

3 *E*_interaction_ = *E*_total_ − *E*_(Fe110-H_2_O-H_3_O-Cl)_ − *E*_inhibitor_

## 3. Results and discussion

### 3.1. Polarization technique

**Fig. 1:**
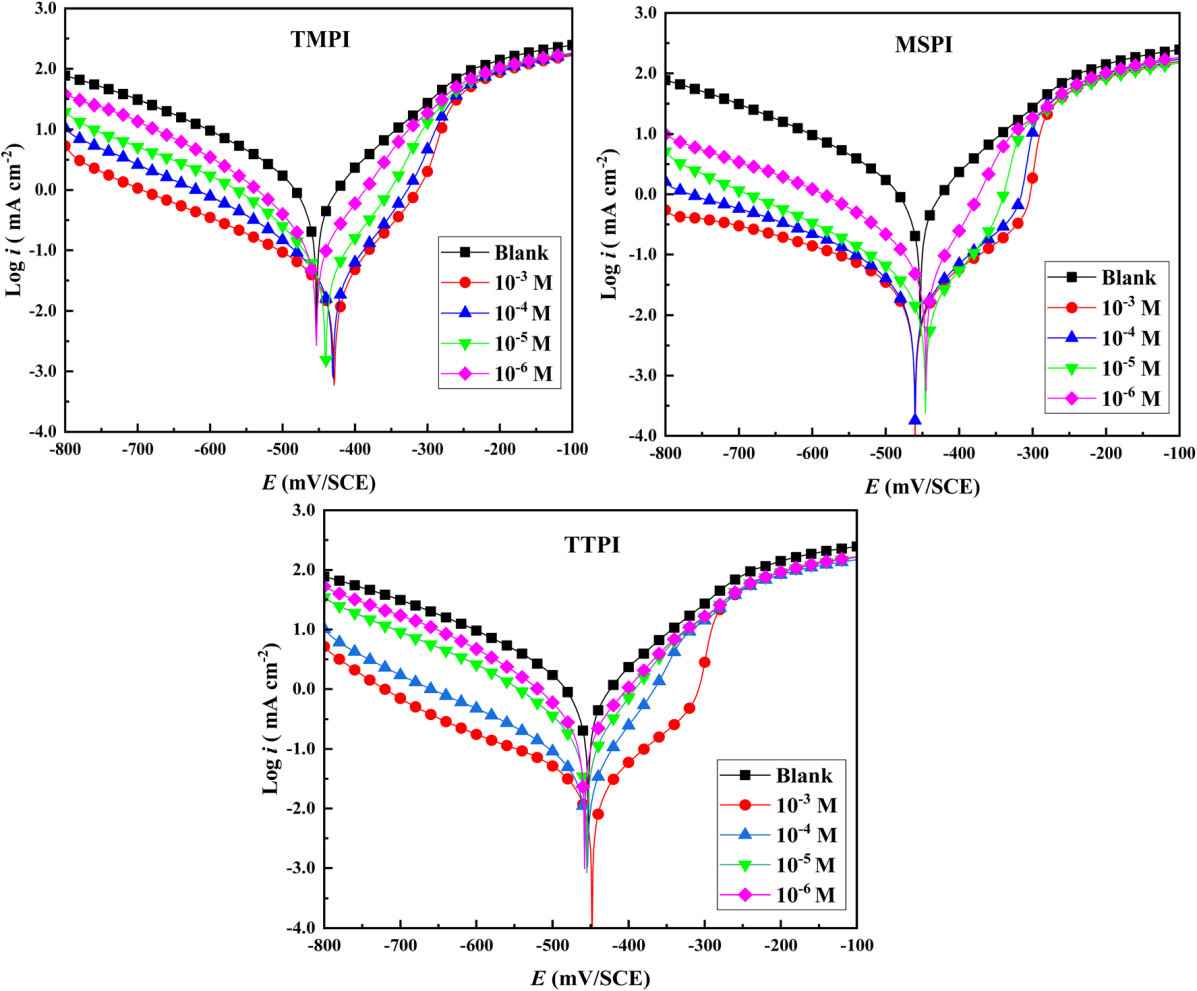
Potentiodynamic polarization curves of carbon steel in 1 M HCl solution without and with different concentrations of dihydroimidazoles

Fig. 1 illustrates the polarization diagrams for carbon steel immersed in 1 M HCl with varying concentrations of dihydroimidazoles at 303 K. As shown, these dihydroimidazoles inhibit both anodic and cathodic reactions, indicating that they prevent the anodic breakdown of carbon steel and slow down hydrogen liberation in cathodic areas.^44^ The Tafel lines of the cathodic branches display similar features after introducing dihydroimidazoles and remain parallel to the reference solution, suggesting that the hydrogen reduction reaction primarily occurs *via* the charge transfer mechanism.^45^ However, the anodic curves of carbon steel in 1 M HCl with dihydroimidazole molecules show no effect at potentials above −280 mV/SCE, where significant steel breakdown occurs, leading to the desorption of the inhibiting layer. In this case, the desorption rate of the dihydroimidazoles surpasses their adsorption rate.^46^ Additionally, Fig. 1 demonstrates that both anodic and cathodic curves are impeded after adding dihydroimidazole molecules to the aggressive solution. These molecules reduce the *i*_corr_, and their effectiveness increases with higher concentrations. However, polarization plots indicated that introducing the three dihydroimidazoles led to only minor alterations in the corrosion potential (*E*_corr_), with shifts not surpassing 28 mV/SCE. Specifically, the TMPI inhibitor caused a slight anodic shift, the MSPI resulted in a minor cathodic shift, and TTPI showed no notable change in *E*_corr_. From an electrochemical perspective, such minimal variations in corrosion potential (less than ±85 mV/SCE) suggest that the dihydroimidazoles do not predominantly influence either the anodic or cathodic reactions independently. Thus, they are categorized as mixed-type inhibitors.^47^ The *i*_corr_ values (Table 2) show a significant reduction when dihydroimidazoles are applied at a concentration of 10^−3^ M in 1 M HCl, specifically reducing from 1104.10 μA cm^−2^ to 15.489 μA cm^−2^ with TMPI, to 9.629 μA cm^−2^ with MSPI, and to 16.196 with TTPI. The calculated *η*_p_ (%) at the maximum concentration for these dihydroimidazoles is reported as 98.59% for TMPI, 99.12% for MSPI, and 98.53% for TTPI, respectively. These findings underscore the substantial inhibitory effect of dihydroimidazoles on carbon steel corrosion in acidic environments. As the concentration of dihydroimidazoles increases, more molecules adsorb onto the active sites at the carbon steel/solution interface, leading to enhanced surface coverage (*θ*) and consequently, augmented *η*_p_ (%). The sequence of corrosion inhibition efficiency (MSPI > TMPI > TTPI) can be attributed to structural and electronic factors that affect adsorption and electron transfer abilities. This is because TTPI has three thiophenyl groups, TMPI has three trimethoxyphenyl groups, and MSPI has three methylthiophenyl substituents. In MSPI, the methylthiophenyl groups combine the polarizability of sulfur with the electron-donating effect of a methyl group. The methyl group boosts sulfur's capacity to donate electrons to the steel surface, enhancing its interaction with the steel. Conversely, methoxyphenyl groups in TMPI donate electrons through resonance but are less polarizable than sulfur. The presence of three methoxy groups may cause steric hindrance, decreasing adsorption density compared to methylthiophenyl groups. Although sulfur atoms in thiophenyl groups in TTPI can donate electrons, the lack of methyl groups results in lower electron density compared to methylthiophenyl groups. Additionally, in MSPI, sulfur-containing groups exhibit a strong affinity for metal surfaces due to their polarizability and ability to form coordinate bonds, whereas the thiophenyl groups in TTPI, despite containing sulfur, do not offer the same level of stabilization as MSPI. Besides, the cathodic Tafel slope (*β*_c_) increased after dihydroimidazoles were added to the 1 M HCl medium, indicating that the examined dihydroimidazoles have a direct influence on the kinetics of hydrogen evolution. Additionally, the *β*_a_ values decreased after the addition of dihydroimidazoles, showing that the anodic reaction is influenced by the presence of dihydroimidazoles. This behavior indicated the formation of a protective film due to the adsorption of the dihydroimidazole molecules on the carbon steel surface, plugging the reactive sites of the carbon steel surface. Moreover, the abundance of nitrogen and oxygen atoms in the dihydroimidazoles can facilitate the formation of the dihydroimidazoles-Fe(ii) complex, constituting a protective barrier that prevents the carbon steel dissolution.

**Table 2:** Polarization parameters of carbon steel in 1 M HCl solution without and with different concentrations of dihydroimidazoles

	*C* (M)	−*E*_corr_ (mV_SCE_)	^*i*^_corr_ (μA cm^−2^)	*β*_a_ (mV dec^−1^)	−*β*_c_ (mV dec^−1^)	*η*_p_ (%)	*θ*
TMPI	00	456.3	1104.1	112.8	155.4	—	—
	0.001	428.3	15.5	59.7	87.6	98.59	0.986
	10^−4^	430.3	22.1	65.1	81.4	97.99	0.980
	10^−5^	439.8	28.9	56.9	61.2	97.38	0.9738
	10^−6^	452.7	53.5	46.2	50.3	95.15	0.951
MSPI	00	456.30	1104.1	112.8	155.4	—	—
	0.001	460.1	9.6	78.7	79.0	99.12	0.991
	10^−4^	459.6	11.1	78.2	75.3	98.99	0.990
	10^−5^	444.8	11.3	66.1	70.8	98.97	0.990
	10^−6^	444.9	15.0	33.7	33.6	98.64	0.980
TTPI	00	456.3	1104.1	112.8	155.4	—	—
	0.001	448.3	16.2	88.3	116.9	98.53	0.985
	10^−4^	454.5	21.6	51.6	76.2	98.04	0.980
	10^−5^	455.1	41.1	36.4	38.0	96.28	0.963
	10^−6^	458.3	65.0	39.5	37.9	94.11	0.941

### 3.2. EIS data

**Fig. 2:**
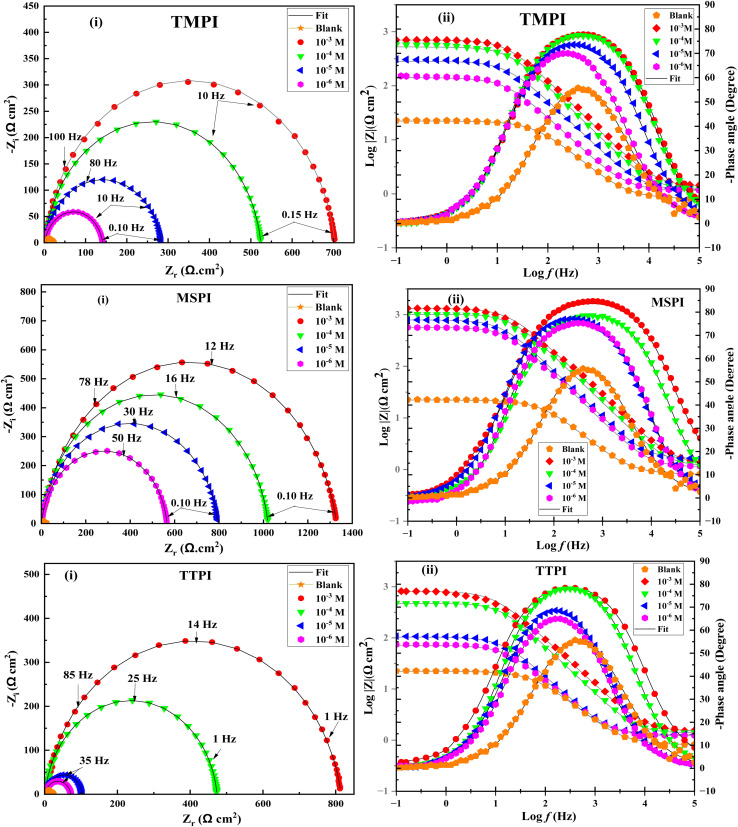
Nyquist (i) and Bode (ii) plots of carbon steel in 1 M HCl solution without and with different concentrations of dihydroimidazoles.

Fig. 2 illustrates the impedance curves of carbon steel in 1 M HCl, both devoid of and in the company of substituted dihydroimidazoles at 303 K, fitted to an equivalent circuit. The compressed and incomplete loops exhibit similar shapes in both the absence and the presence of substituted dihydroimidazoles, indicating that the corrosion mechanism remains unchanged. This phenomenon results from variations in metal surface uniformity and frequency dispersion at the interface.^48^ Additionally, the depressed capacitance circle suggests that charge transfer at the interface is the primary factor in corrosion. Compared to the untreated medium, the size of the semi-circles significantly increases, demonstrating effective blockage of the charge transfer process.^49^ As more substituted dihydroimidazoles are added, the diameter of the loops expands. This observation suggests the formation of the dihydroimidazole adsorption layer on the carbon steel, substantially reducing corrosion. These findings collectively demonstrate the exceptional anti-corrosion capabilities of substituted dihydroimidazoles for carbon steel.

**Fig. 3:**
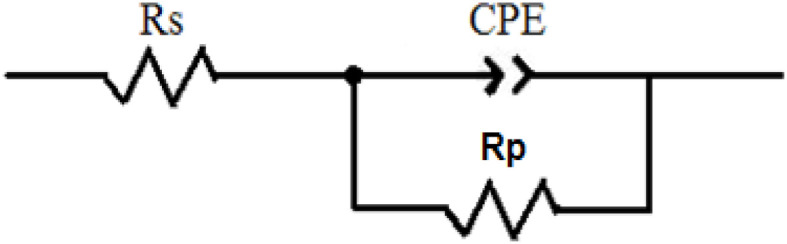
Schematic equivalent circuit standard for modeling the EIS upshots.

Fig. 2(ii) exhibits the Bode plots for carbon steel in 1 M HCl, both devoid of and in the company of dihydroimidazoles, offering a more thorough insight into the corrosion mitigation process. The phase-frequency characteristic curve exhibits a single-phase peak, displaying a one-time constant behavior and the existence of double electric layers.^50^ The phase angle, which is inferior to 90°, implies that it is not an ideal capacitor, necessitating the use of CPE rather than an ideal capacitor to fit the equivalent circuit.^51^ As illustrated in Fig. 3, CPE accurately elucidates interfacial adsorption, roughness, and other interfacial phenomena. These observations indicate that higher dihydroimidazole concentrations enhance adsorption and improve the stability of the formed layer.^52^ The peak value rises significantly with the addition of different dihydroimidazole concentrations compared to the untreated medium, while the frequency range expands considerably, suggesting the slow formation of the dihydroimidazole adsorption film. The modulus–frequency relationship curve in Fig. 2(ii) demonstrates that impedance modulus values at low frequencies rise with increasing dihydroimidazole concentrations, indicating that higher concentrations provide better corrosion prevention. Compared to the untreated medium, impedance modulus (log|*Z*|) values rise by 1 to 1.8 orders of magnitude at optimal dihydroimidazole concentrations, confirming the adsorption of dihydroimidazole molecules on carbon steel and effectively inhibiting corrosion from aggressive acid.^53^ The log-linear relationship between frequency and impedance modulus has a slope close to −1, affirming the typical electric capacity property of the homogeneous dihydroimidazole-adsorption layer.^54^ The similar profiles in Fig. 2 denote the same corrosion inhibition mechanism.

Table 3 displays fitted findings generated from the circuit equivalent of Fig. 3, including solution resistance (*R*_s_), polarization resistance (*R*_p_), exponent and constant of CPE (*n*, *Q*), double layer capacitance (*C*_dl_), and shielding effectiveness (*η*_imp_ (%)). The CPE's fitting impedance is computed *via* the equation given below.^55^

4 *Z*_CPE_ = *Q*^−1^(*iω*)^−*n*^

where *ω* and *i* denote the angular frequency and the imaginary unit, respectively,

**Table 3:** EIS parameters of carbon steel in 1 M HCl solution without and with different concentrations of dihydroimidazoles

	C (M)	*R*_s_ (Ω cm^2^)	*R*_p_ (Ω cm^2^)	10^6^ × *Q* (μF s^*n*−1^ cm^−2^)	*n*	*C*_dl_ (μF cm^−2^)	*χ* ^2^	*η*_imp_ (%)
TMPI	00	0.83	21.57	293.9	0.845	116.2	0.002	—
	0.001	1.38	701.7	21.124	0.911	13.99	0.009	96.92
	10^−4^	1.193	522.9	26.731	0.911	17.61	0.009	95.87
	10^−5^	1.161	282.7	57.036	0.894	34.96	0.009	92.37
	10^−6^	1.146	140.4	122.16	0.885	72.03	0.008	84.63
MSPI	00	0.83	21.57	293.9	0.845	116.2	0.002	—
	0.001	1.249	1328	16.342	0.940	9.645	0.009	98.37
	10^−4^	1.153	1019	17.048	0.903	11.03	0.009	97.88
	10^−5^	1.626	793.4	33.307	0.901	23.56	0.009	97.28
	10^−6^	1.133	564.5	36.224	0.900	24.89	0.009	96.17
TTPI	00	0.83	21.57	293.9	0.845	116.2	0.002	—
	0.001	1.495	813	31.141	0.936	20.60	0.009	97.35
	10^−4^	1.377	472.1	34.93	0.933	26.01	0.009	95.43
	10^−5^	1.278	102.5	170.69	0.897	107.26	0.008	78.95
	10^−6^	1.217	70.88	224.48	0.880	127.63	0.007	69.56

However, the *C*_dl_ is given by the next equation.^55^5
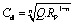


The addition of substituted dihydroimidazoles notably enhances the *R*_p_ and reduces the *C*_dl_, an effect that becomes more pronounced with increasing dihydroimidazole concentrations. These observations suggest that dihydroimidazole molecules spontaneously adsorb onto the steel/acid interface, forming a surface film that impedes charge transfer.^56^ Typically, the double electric layer capacitance is influenced by factors such as dielectric constant, protected area, and thickness.^57^ The reduction in *C*_dl_ value occurs because corrosion inhibitors with lower dielectric constants can displace adsorbed water molecules on the surface, creating a protective film and increasing the electronic double-layer thickness.^58^ Furthermore, the *n* values ranging from 0.84 to 0.940 approach 1, indicating surface homogeneity of the steel devoid of and in the company of substituted dihydroimidazoles and suggesting that the charge transfer process governs the dissolution mechanism of carbon steel in the test solution.^59^ The variation in *n* values with increasing inhibitor concentration may be attributed to the adsorption of dihydroimidazole molecules. This implies that substituted dihydroimidazole molecules interact with the steel surface, gradually replacing H_2_O molecules at the steel/solution interface.^60^ Additionally, *Q*, which describes the fluency of load charge through the conductor and is the reciprocal of impedance, decreases significantly when the sample is immersed in HCl with various concentrations of substituted dihydroimidazoles. This suggests that the interface structure is modified due to the adsorption of dihydroimidazole molecules. Chi-square (*χ*^2^) was adopted to assess the goodness of the fit for the EIS data. All results provided low *χ*^2^ values of around 0.001 (Table 3), reflecting that the fitting data were well consistent with the experimental results. The *η*_imp_ (%) values rise with increasing substituted dihydroimidazoles concentration, and the order is TTPI < TMPI < MSPI, demonstrating a trend where inhibition potency rises with the three substituted alkyl groups in the imidazole ring. MSPI's superior performance is due to its optimal combination of electron-rich sulfur, methyl-enhanced electron donation (methylthiophenyl groups), and effective surface packing. These substituents also increase hydrophobicity and improve surface coverage, minimizing metal exposure to corrosive ions.

### 3.3. Isotherm of adsorption models

Previous studies have shown that inhibitors reduce metal corrosion by attaching to the metal surface and filling active sites. This research employs various adsorption isotherm models, such as Langmuir, Frumkin, Temkin, and Freundlich, to fit experimental data and investigate the interactions between substituted dihydroimidazoles and the carbon steel surface. These models are described by the following equations:^61^

6 Langmuir: *C*(*θ*)^−1^ = (*K*_ads_)^−1^ + *C*

7 Frumkin: ln *θ*((1 − *θ*) × *C*)^−1^ = −ln *K*_ads_ + 2*fθ*

8 Temkin: exp(*fθ*) = *K*_ads_*C*

9 Freundlich: ln *θ* = ln *K*_ads_ + *f*ln *C*

In the given formulas, *θ* describes the surface coverage (*η*_p_ (%)/100), and *K*_ads_ denotes the adsorption equilibrium constant.

**Fig. 4:**
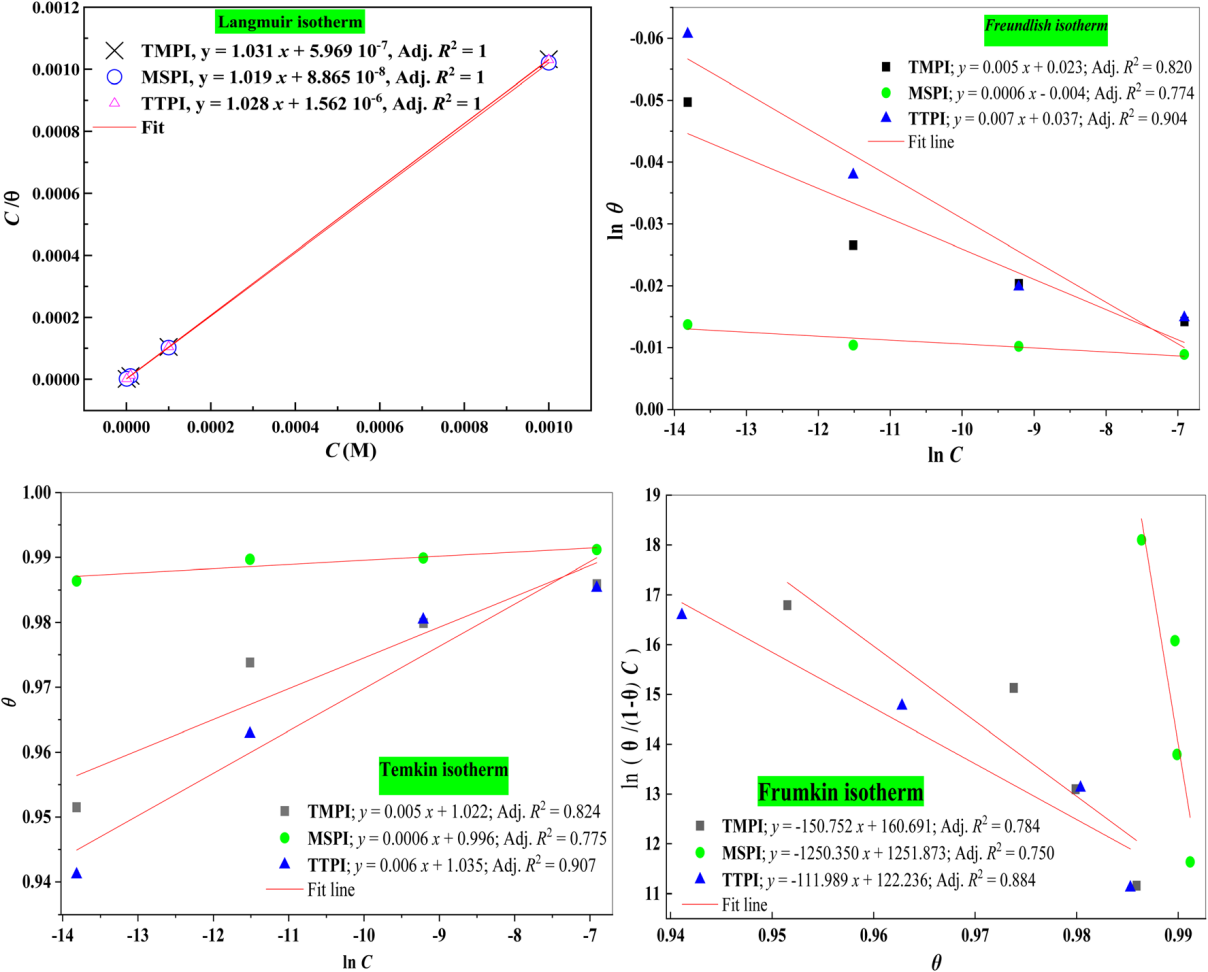
Adsorption isotherm models for carbon steel in 1 M HCl solution with different concentrations of dihydroimidazoles.

Fig. 4 demonstrates the function diagram corresponding to eqn (6)–(9). Through observation, the Langmuir adsorption calculated by eqn (6) showed the best fit with slopes and the correlation coefficient (*R*^2^) values very close to unity, indicating that the experimental data were consistent with the Langmuir isotherm. Eqn (10) was used to calculate the standard adsorption free energy (Δ*G*_ads_) values:^62^10
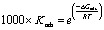


Table 4 shows high *K*_ads_ values, suggesting effective and spontaneous adsorption of substituted dihydroimidazoles onto metal surfaces. Meanwhile, the negative Δ*G*_ads_ values suggest that the adsorption of substituted dihydroimidazoles on carbon steel surfaces is spontaneous.

**Table 4:** Thermodynamic parameters of the adsorption of dihydroimidazoles in 1 M HCl solution

	*R* ^2^	Slope	*K*_ads_ (L mol^−1^)	Δ*G*_ads_ (kJ mol^−1^)
TMPI	1	1.07	9.3 10^4^	−38.91
MSPI	1	1.03	4.71 10^6^	−48.81
TTPI	1	1.2	7.1 10^4^	−38.24

### 3.4. Corrosion temperature study

**Fig. 5:**
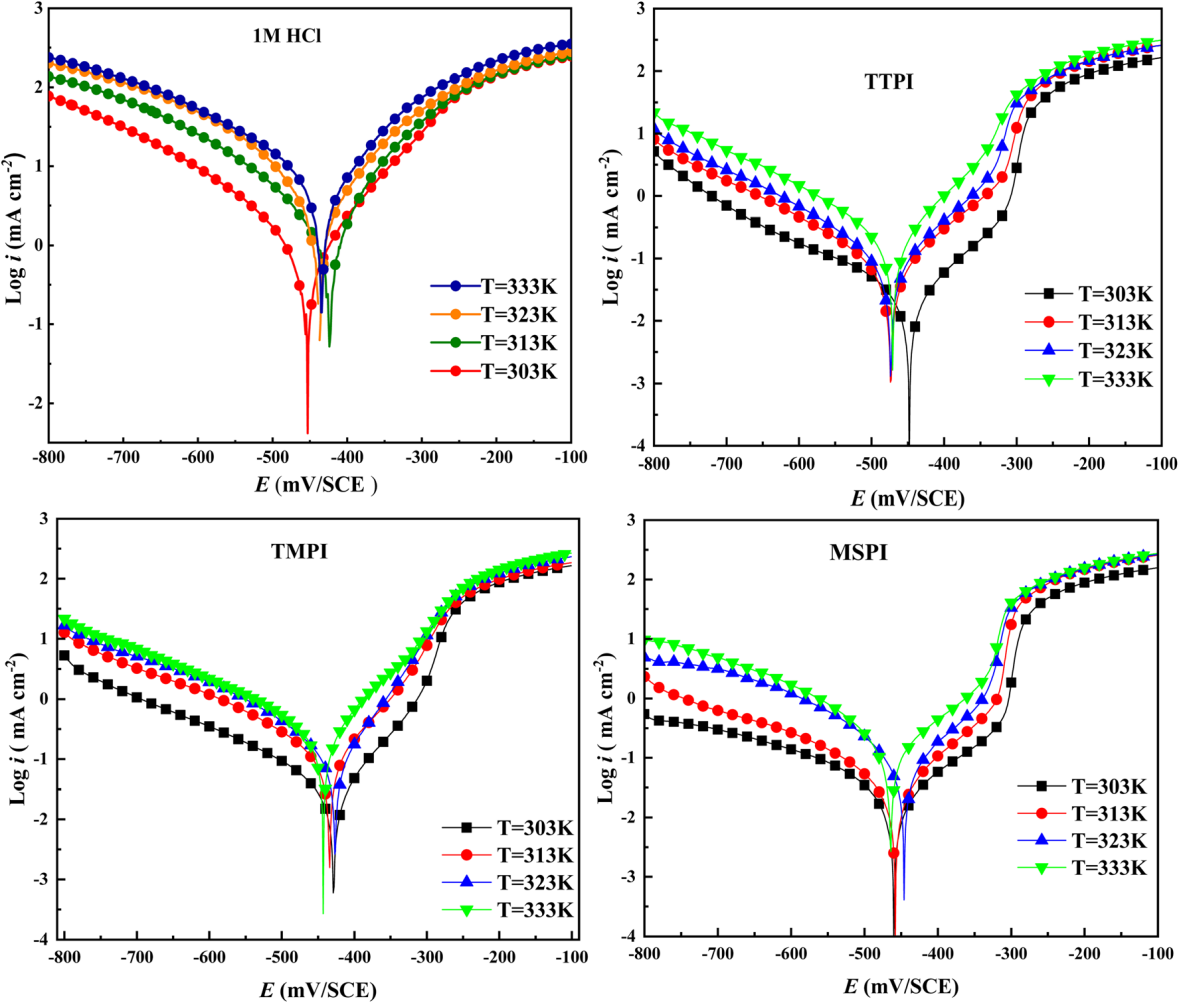
Potentiodynamic polarization curves of carbon steel in 1 M HCl solution without and with 10^−3^ M of dihydroimidazoles at various temperatures.

Temperature may affect both the material's behavior in a corrosive environment and the metal-dihydroimidazole interaction. To better understand the mechanism of inhibition by calculating the thermodynamic energies of the corrosion process, it is necessary to analyze the effect of temperature on the degree of protection. The polarization technique was used to study the impact of temperature on our inhibitor's ability to prevent carbon steel corrosion at 303, 313, 323, and 333 K, with and without dihydroimidazoles at 0.001 M. As seen in Table 5 and Fig. 5, without any dihydroimidazoles, the *i*_corr_ is 10 125 (μA cm^−2^) at 303 K, which rises sharply to 3944.9 (μA cm^−2^) at 333 K. This significant enlargement in *i*_corr_ implies that the carbon steel becomes more vulnerable to HCl attack at elevated temperatures. The introduction of dihydroimidazoles results in a slight boost in *i*_corr_ across all temperatures compared to when they are absent, signifying their ability to protect the carbon steel surface. However, their protective effect remains consistent as the temperature rises, implying that they maintain their efficacy as dihydroimidazoles with increasing temperatures. These findings imply that dihydroimidazoles exhibit temperature independence.

**Table 5:** Electrochemical parameters of carbon steel in 1 M HCl solution without and with 10^−3^ M of dihydroimidazoles at various temperatures

	*T* (K)	−*E*_corr_ (mV SCE)	*i*_corr_ (μA cm^−2^)	*β*_a_ (mV dec^−1^)	-*β*_c_ (mV dec^−1^)	*η*_p_ (%)
1 M HCl	303	456.3	1104.1	112.8	155.4	—
	313	423.5	1477.4	91.3	131.3	—
	323	436.3	2254.0	91.4	117.8	—
	333	433.3	3944.9	103.9	134.6	—
0.001 M of TMPI	303	428.256	15.489	59.7	87.6	98.59
	313	433.131	35.823	41.6	63.8	97.57
	323	426.962	44.977	51.4	58.4	98.00
	333	443.016	61.222	37.3	43.5	98.44
0.001 M of MSPI	303	460.092	9.629	78.7	79.0	99.12
	313	458.110	14.060	66.1	81.6	99.04
	323	445.498	24.617	49.6	49.4	98.90
	333	464.482	42.765	49.4	44.7	98.91
0.001 M of TTPI	303	448.314	16.196	88.3	116.9	98.53
	313	473.238	34.201	74.7	94.0	97.68
	323	473.265	46.672	78.8	93.9	97.92
	333	471.246	88.378	66.7	81.1	97.75

**Fig. 6:**
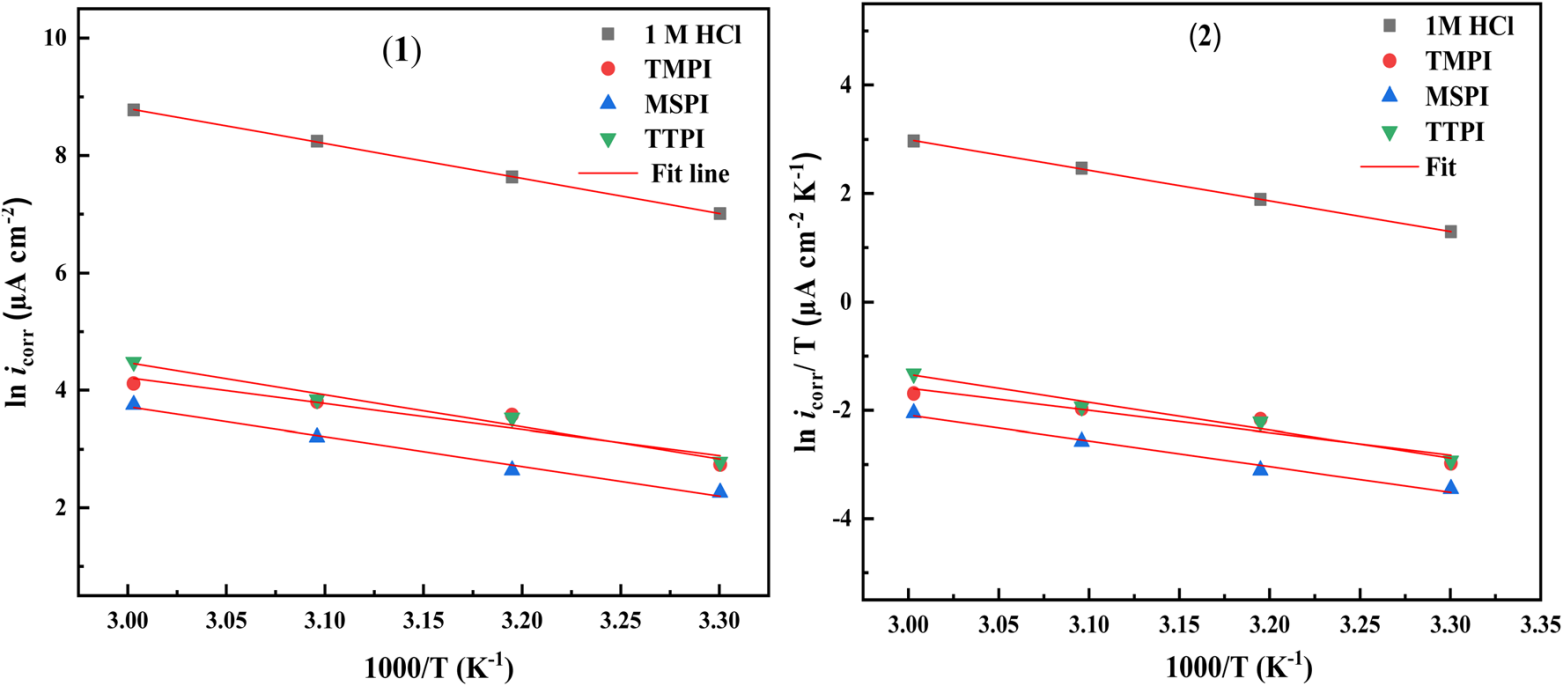
Arrhenius (1) and transition state (2) plots of carbon steel in 1 M HCl solution without and with 10^−3^ M of dihydroimidazoles at various temperatures.

The Arrhenius and transition state diagrams for systems in 1 M HCl containing dihydroimidazoles at various temperatures are shown in Fig. 6 (1)/(2). Consequently, the relevant activation parameters derived from the slopes and intercepts of the fitting lines are included in Table 6, encompassing the apparent activation energy (*E*_a_), the enthalpy (Δ*H*_a_), and the entropy (Δ*S*_a_). The relationship between temperature and corrosion rate is examined using the Arrhenius and transition state eqn (11) and (12):^63^11
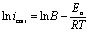
12
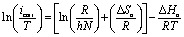
In the previous equations, *R* (8.314 J mol^−1^ K^−1^), *B* (pre-exponential factor), *N* (Avogadro's number, 6.022 × 10^23^ mol^−1^), and *h* (Planck's constant, 6.626 × 10^−34^ J s) denoted the gas constant, pre-exponential factor, Avogadro's number, and Planck's constant, respectively.

**Table 6:** Activation parameters for the carbon steel dissolution in 1 M HCl without and with dihydroimidazoles

	*E*_a_ (kJ mol^−1^)	Δ*H*_a_ (kJ mol^−1^)	Δ*S*_a_ (J mol^−1^ K^−1^)
1 M HCl	35.43	32.79	−79.22
TMPI	36.74	34.1025	108.36
MSPI	42.08	39.44	96.48
TTPI	45.32	42.69	80.51

The *E*_a_ value is higher in the system with dihydroimidazoles compared to the system without, indicating that the corrosion process must overcome a greater energy barrier due to adsorption.^64^ The positive Δ*H*_a_ value signifies that metal dissolution in HCl is a slow endothermic process, with higher temperatures supplying more energy to accelerate metal degradation.^65^ The negative Δ*S*_a_ value suggests that the slow formation of an activated complex from metal atoms controls the corrosion process, rather than diffusion and dissociation. The positive Δ*S*_a_ value indicates that dihydroimidazoles interact with surface metal atoms to create an adsorption film, disrupting the transition state reaction of corrosion and altering the reaction path to reduce the corrosion rate.^66^ Compared to the 1 M HCl, there is a trend of increasing Δ*S*_a_ in the presence of dihydroimidazoles, as they impede the formation of active complexes and increase system chaos through adsorption, implying that corrosion becomes more difficult.

### 3.5. SEM-EDS study

**Fig. 7:**
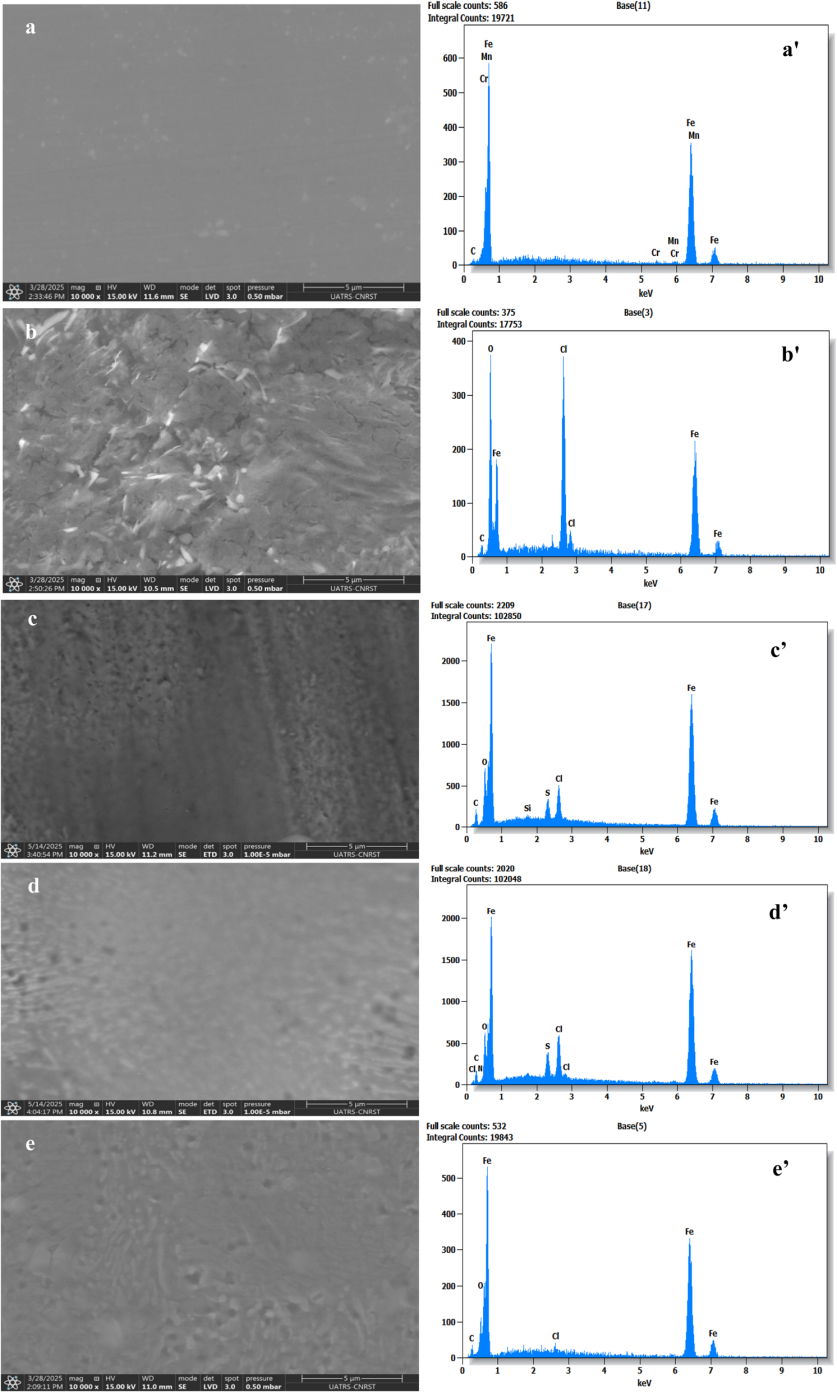
SEM-EDS photographs acquired for the carbon steel surface: after its polishing (a–a′), 6 hours after its soaking in 1 M HCl (b–b′), and 6 hours after its soaking in 1 M HCl with 10^−3^ M of TMPI (c–c′), TTPI (d–d′), and MSPI (e–e′).

The surface morphologies of carbon steel were examined utilizing SEM-EDS both before and after a 6-hours immersion in 1 M HCl with an addition of 10^−3^ M of various dihydroimidazoles to understand the corrosion inhibition mechanism of the studied dihydroimidazoles. Fig. 7a shows the SEM image of the polished carbon steel surface, which appears to have a homogeneous surface morphology and is relatively smooth. In the 1 M HCl solution without dihydroimidazoles, a large amount of corrosion products appeared (Fig. 7b), and the surface was significantly rougher than in its polished state. After adding 10^−3^ M of dihydroimidazoles, the corrosion products on the carbon steel surface were greatly reduced, and the surface products appeared more like a thin film (Fig. 7c–e), which was obviously smoother than the metal surface soaked in the free HCl solution for 6 hours.

The EDS results are displayed in Table 7 and Fig. 7a′–e′. Fig. 7a′ illustrates that the primary constituents of carbon steel are Fe and C. Following six hours of soaking in a 1 M HCl solution (Fig. 7b′), the metal surface's Fe atom content fell precipitously from 95.5 at% to 39.1 at%, its O content rose from 0 at% to 38.9 at%, and Cl atoms surfaced (17 at%), all of which indicated severe metal corrosion. After adding dihydroimidazoles, the Cl content dropped significantly (from 17.0 at% to 2.3 at% TMPI, 2.9 at% for TTPI, and 1.2 at% for MSPI) in comparison to the metal in 1 M HCl solution (Fig. 7c′–e′). This is because dihydroimidazoles create a thick protective layer on the carbon steel's surface, preventing the HCl solution from dissolving it.

**Table 7:** EDS data for carbon steel before and after 6 hours of soaking in 1 M HCl, without and with 10^−3^ M of dihydroimidazoles

Element	Condition									
	Carbon steel		Dipped carbon steel in 1 M HCl		TMPI		TTPI		MSPI	
	Weight (%)	Atom (%)	Weight (%)	Atom (%)	Weight (%)	Atom (%)	Weight (%)	Atom (%)	Weight (%)	Atom (%)
C	0.6	2.6	1.8	5.1	1.2	3.0	1.1	2.6	1.5	6.0
N	—	—	—	—	—	—	0.2	0.3	—	—
Cr	0.5	0.5	—	—	—	—	—	—	—	—
Mn	1.4	1.4	—	—	—	—	—	—	—	—
O	—	—	17.9	38.9	32.3	60.0	32.3	59.9	2.9	8.9
Si	—	—	—	—	0.2	0.2	—	—	—	—
S	—	—	—	—	1.4	1.3	1.8	1.7	—	—
Cl	—	—	17.4	17.0	2.7	2.3	3.5	2.9	0.8	1.2
Fe	97.5	95.5	62.9	39.1	62.2	33.2	61.2	32.6	94.8	84.0
Total	100	100	100	100	100	100	100	100	100	100

### 3.6. Contact angle analysis

**Fig. 8:**
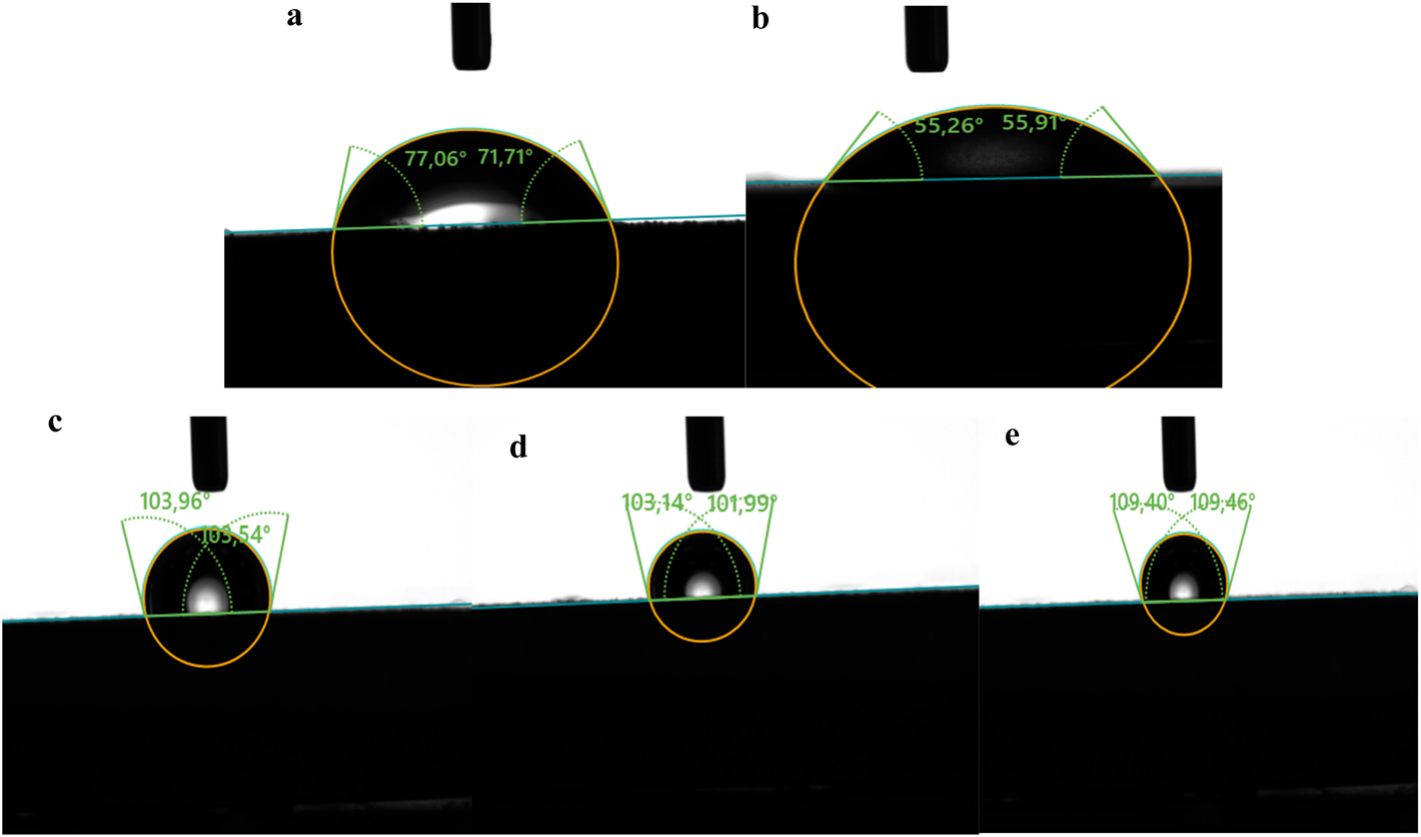
Water contact angle results for polished carbon steel (a) and after immersion in 1 M HCl without (b) and with 10^−3^ M of TMPI (c), TTPI (d), and MSPI (e).

Studies exhibit that surface with water contact angles exceeding 90° exhibit hydrophobic properties, whereas metallic substrates with angles below 90° are hydrophilic.^67^Fig. 8 illustrates the water contact angles of carbon steel samples subjected to 1 M HCl for 6 hours, both with and without dihydroimidazoles. The polished carbon steel sample shows a contact angle of 74.38° (Fig. 8a), while the sample immersed in 1 M HCl has an angle of 55.58° (Fig. 8b), indicating the hydrophilic nature of the steel surface. The reduced contact angle results from corrosive ions in the solution attacking the submerged carbon steel surfaces, leading to accelerated surface degradation. This corrosion causes water droplets to spread across the surface, creating wetness. Conversely, the introduction of TMPI, MSPI, and TTPI increases the water contact angles of carbon steel from 55.58° (1 M HCl) to 109° (MSPI), 104° (TMPI), and 103° (TTPI), respectively. These values, all above 90°, suggest that dihydroimidazoles adsorb onto carbon steel surfaces, protecting them from Cl^−^ ion attacks and enhancing water repellence. The observed sequence of water contact angle values (MSPI > TMPI > TTPI) aligns with the experimentally determined order of corrosion inhibition effectiveness. The methylthiophenyl groups in MSPI enhance hydrophobicity due to their non-polar methyl and sulfur components, resulting in a denser and more protective adsorption layer. In contrast, trimethoxyphenyl groups in TMPI, although electron-rich, contain oxygen atoms that may interact with water, reducing their overall hydrophobicity compared to methylthiophenyl groups. The thiophenyl groups in TTPI lack both the additional electron-donating and hydrophobic effects, making them the least effective.

### 3.7. XRD analysis

**Fig. 9:**
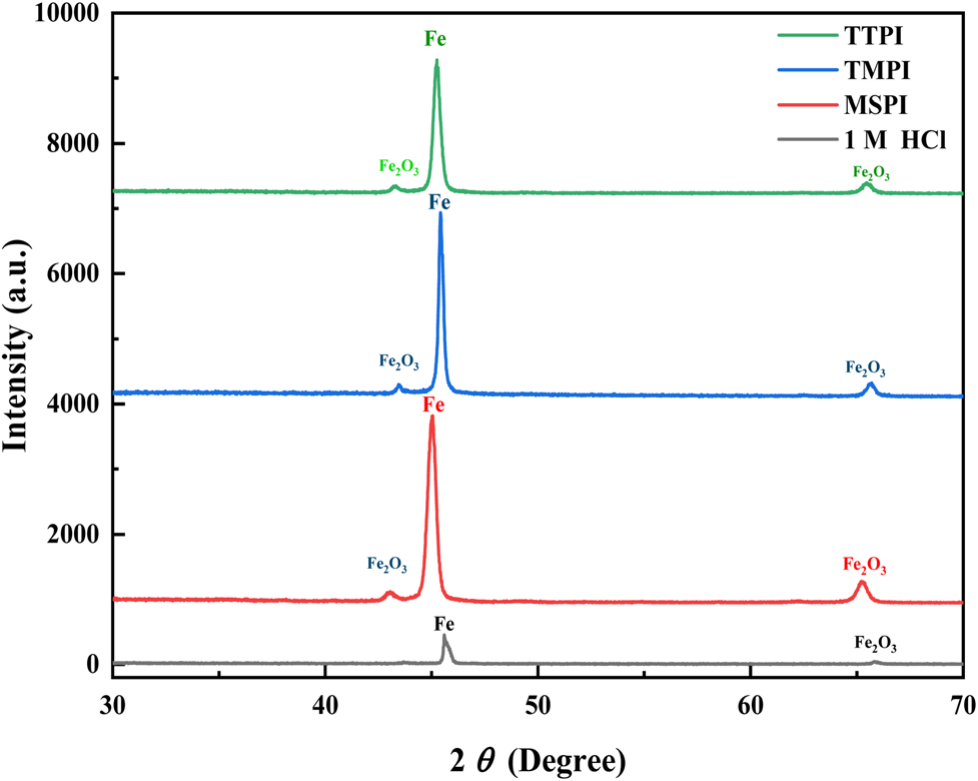
XRD patterns for immersed carbon steel in 1 M HCl without and with 10^−3^ M of dihydroimidazoles.

XRD has been considered an attractive option for identifying the crystalline phase and determining the nature of the protective layer formed on the metallic surface. Fig. 9 demonstrates the X-ray diffraction patterns of the scratched specimens after 6 hours of soaking in 1 M HCl alone and including 0.001 M dihydroimidazoles. The graph demonstrates two peaks at 2*θ* = 45.63° and 2*θ* = 79.13° attributable to Fe, as well as two peaks at 45° and 65.70° due to iron oxide (Fe_2_O_3_).^68^ Careful examination (Fig. 9) revealed that the XRD patterns of scratched specimens containing dihydroimidazoles had XRD peaks with high intensities. The rise in XRD pattern intensities reveals that the nature of the formed protective oxide layers is non-crystalline, supports the development of a protective layer, and has a greater degree of anticorrosion when dihydroimidazoles are present.

### 3.8. AFM analysis

**Fig. 10:**
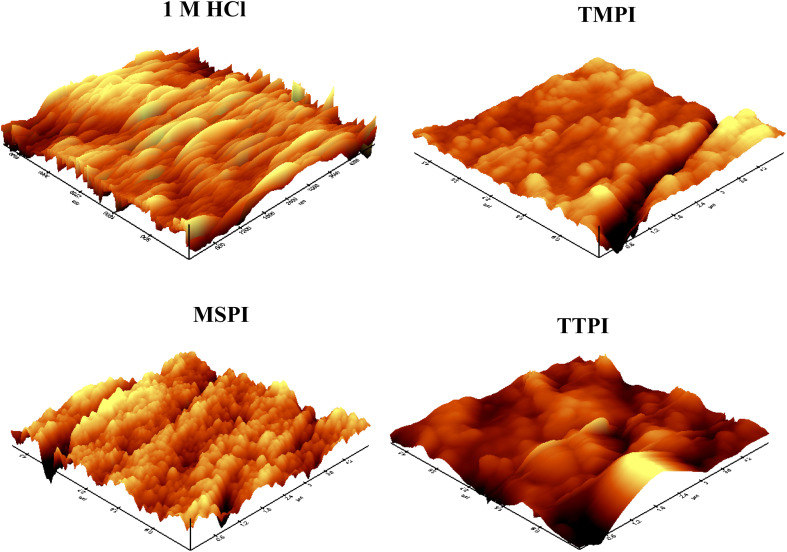
AFM micrographs of carbon steel surface in 1 M HCl without and with 10^−3^ M of dihydroimidazoles.

Fig. 10 shows the AFM picture of a steel surface after a dip in 1 M HCl, free of 0.001 M dihydroimidazoles and containing them. As viewed in Fig. 10a, being exposed to 1 M HCl significantly degraded the steel surface, resulting in an extremely rough texture. Fig. 10b and c depicts the carbon steel surface treated with dihydroimidazoles, which is extremely smooth and homogeneous. The addition of dihydroimidazoles to 1 M HCl causes a considerable reduction in surface roughness, suggesting that chlorine ions have limited access to the steel surface. This demonstrates the efficiency of the dihydroimidazole layer in preserving steel in HCl.^69^

### 3.9. Quantum chemical calculations

#### 3.9.1. Geometries and energies

**Fig. 11:**
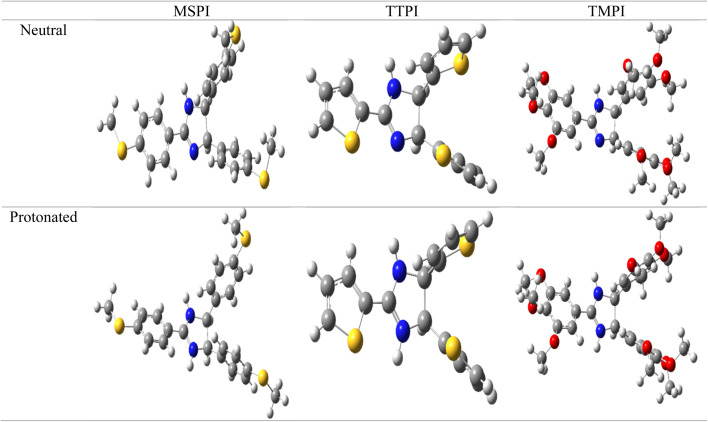
Optimized molecular structures (neutral and protonated) of the three corrosion inhibitors: TMPI, MSPI, and TTPI at the B3LYP/6-311G(d,p) level of theory.

The three molecules studied, designated by MSPI, TMPI, and TTPI, showed potential inhibitory efficiency against corrosion. Thus, to analyze their electronic and structural properties and establish a link between their structure and their inhibitory efficiency, an optimization of the geometries was carried out by quantum processing using the DFT method and the B3LYP functional at the 6-311G(d,p) level. The optimized geometric structures of these three molecules are proposed in Fig. 11.

MSPI, TMPI, and TTPI consist of a central dihydroimidazole ring substituted by thiazole (thiophenyl) rings (for TTPI), phenyl rings functionalized by methylthio groups (for MSPI), and phenyl rings, each fixing a methoxy group (for TMPI).

After geometric optimization, the three studied molecules present conjugated systems and the lone pair on the N, O, and S heteroatoms, which favors their effective adsorption capacity. Likewise, their specific structural arrangements influence their reactivity and their interaction with the metal surface. The three compounds are characterized by a partial flatness between the central imidazole core and one of the substituted rings, while the other rings deviate from the plane.

For TTPI, the central dihydroimidazole ring and thiophene rings are located in the same plane, while the other two imidazole rings are shifted out of this plane, creating a molecular distortion. For MSPI and TMPI, the arrangement is similar; one of the three substituted phenyl rings remains aligned with the central dihydroimidazole ring, while the other two rings are oriented out of the main plane, adopting an inclined orientation relative to the central plane. This molecular arrangement can influence the way in which the inhibitors interact with the metal surface, in particular by favoring certain adsorption sites depending on the electronic distribution and the functional groups present.

#### 3.9.2. Quantum chemical descriptors

The MSPI, TMPI, and TTPI compounds have demonstrated excellent inhibitory efficacy, exceeding 97%. These experimental results are corroborated and justified by our theoretical calculations, highlighting the electronic and structural properties at the origin of their performance. In order to establish a relationship between the inhibition efficiency and the optimized structure of the studied molecules, we calculated the necessary quantum reactivity parameters. The values of the energy parameters obtained at the same calculation level of theory are listed in Table 8.

**Table 8:** The quantum chemical parameters of the three dihydroimidazoles

*E*	HOMO	LUMO	*E*_g_	*I*	*A*	*χ*	*η*	*μ*	Dipole moment (D)	*σ*	*ω*	Δ*E*_back-donation_	Δ*N*
MSPI	−5.892	−1.293	4.600	5.892	1.293	3.593	2.300	−3.593	5.851	0.435	2.806	−0.575	0.267
TMPI	−5.874	−1.085	4.789	5.874	1.085	3.479	2.395	−3.479	1.053	0.418	2.528	−0.599	0.280
TTPI	−6.091	−1.494	4.597	6.091	1.494	3.793	2.299	−3.793	2.867	0.435	3.129	−0.575	0.223
MSPI-H	−8.142	−5.263	2.879	8.142	5.263	6.702	1.440	−6.702	8.128	0.695	15.601	−0.360	−0.654
TMPI-H	−8.387	−5.034	3.352	8.387	5.034	6.710	1.676	−6.710	7.291	0.597	13.432	−0.419	−0.564
TTPI-H	−9.517	−5.906	3.610	9.517	5.906	7.712	1.805	−7.712	5.584	0.554	16.472	−0.451	−0.801

**Fig. 12:**
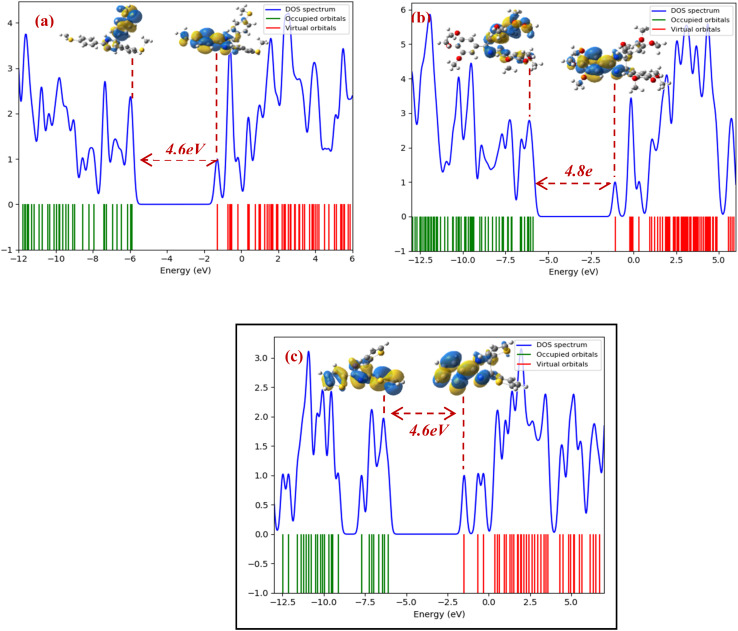
DOS spectra and HOMO–LUMO orbitals of (a) MSPI, (b) TMPI, and (c) TTPI molecules.

According to the calculated values of the OMF energies (Fig. 12), it is noted that the three molecules show high values of the energy of the HOMO orbital (*E*_HOMO_ = −5.892, −5.874, and −6.091 eV for MSPI, TMPI, and TTPI, respectively), hence a higher efficiency of the inhibition of corrosion.^70^ Indeed, these high values reflect a greater capacity of the electron-rich centers of the inhibitor to donate electrons to the vacant d orbitals of the Fe metal. Likewise, the displayed values of the energies of the LUMO orbital (*E*_LUMO_ = −1.293, −1.085, and −1.494 eV for MSPI, TMPI, and TTPI, respectively) indicate a high capacity of these inhibitors to accept electrons, which improves their efficiency by facilitating electronic exchange with the d occupied orbitals.

The values of the energy gap (Δ*E*) of the three molecules, located around 4.6 eV for molecule MSPI and molecule TTPI and 4.8 eV for molecule TMPI, indicate a similar chemical reactivity, but with subtle differences. Molecules MSPI and TMPI, with the lowest energy gap (Δ*E*), have a slightly higher reactivity, followed by TMPI molecules. A lower energy gap is generally associated with better corrosion inhibition efficiency and with more intense adsorption of the inhibitory molecules on the metal surface.^71^ These results indicate that molecules MSPI and TTPI are the most favorable, compared to molecule TMPI, for strong chemical interactions, such as adsorption on the carbon steel surface. This conclusion is in perfect agreement with the experimental data obtained, thus confirming their superior performance as corrosion inhibitors.

In an acidic medium, the protonation of MSPI, TMPI, and TTPI modifies their electronic characteristics and increases their reactivity. Indeed, it leads to a reduction in the energy gap, thus making the molecules more reactive. The energies of the HOMO orbitals decrease compared to the neutral state, which reduces their potential to donate electrons. Simultaneously, the LUMO orbitals become lower in energy, which strengthens their ability to attract electrons.

In accordance with Pearson's principle,^72^ molecules having a high global softness (*σ*) and a low global hardness (*η*) favor increased inhibition. The softness values (0.435 eV for molecule MSPI and molecule TTPI and 0.418 eV for molecule TMPI) show that molecules MSPI and TTPI have similar adsorption capacities, slightly greater than that of molecule TMPI. This confirms that molecules MSPI and TTPI, thanks to their increased softness, interact more effectively with the metallic surface. Concerning electronegativity (*χ*), molecule TMPI (*χ* = 3.479 eV) is the least electronegative due to the OCH_3_ groups, which are strong electron donors by mesomerism. This promotes a transfer of electrons to the metal surface, improving adsorption. Molecule MSPI (*χ* = 3.593 eV), with its SCH_3_ groups, exhibits intermediate electronegativity, combining a moderate inductive attractor effect and a weak donor effect. Molecule TTPI, on the other hand, is the most electronegative (*χ* = 3.793 eV) due to the thiophenyl, which stabilizes the positive charges and increases the electrophilicity (*ω* = 3.129 eV). On the other hand, molecule TMPI, with its OCH_3_ groups, is the least electrophilic (*ω* = 2.528 eV), because these groups reduce its ability to accept electrons.

The electron back-donation (Δ*E*_back-donation_) is higher for molecule TMPI (−0.599), thanks to the OCH_3_ groups, which favor the retro-donation. Molecules MSPI and TTPI have slightly lower electron back-donation values (−0.575) but remain competitive. The SCH_3_ groups and the thiophenes also have donor effects, although less pronounced than those of the OCH_3_. The positive values of the electron transfer fraction Δ*N* confirm that the electrons are transferred from the inhibitory molecules to the iron surface, thus reinforcing the protection against corrosion. Molecule TMPI (Δ*N* = 0.280) gives up its electrons more easily than molecule MSPI (Δ*N* = 0.267) and molecule TTPI (Δ*N* = 0.223). However, in terms of dipole moment, molecules MSPI (5.851 D) and TTPI (2.867 D) are significantly more polar than molecule TMPI (1.053 D). This increased polarity allows molecules MSPI and TTPI to form stronger bonds with the active sites of the metal, favoring more stable adsorption and a more effective protective layer.

Although molecule TMPI is distinguished by its strong ability to donate electrons and its high electron back-donation, molecules MSPI and TTPI, thanks to their increased polarity, their enhanced electrostatic interactions, and their superior chemical reactivity, offer optimal protection against corrosion. These results underline the importance of the electronic and structural effects of the substituents, with a predominance of sulfur-containing groups (SCH_3_ and thiophenes) for an effective inhibition of corrosion.

Protonation makes molecules more electrophilic, facilitating their interaction with the electron-rich d orbitals of the Fe metal. There is also an increase in electronegativity (6.7 eV for MSPI-H and TI and 7.7 eV for TMPI-H), which indicates that protonated molecules attract electrons more strongly. This stabilizes them in electron-rich media. In addition, protonated molecules have a high softness and a reduced hardness (Table 8), which makes them more polarizable and less stable than their neutral forms. Thus, once protonated, the MSPI, TMPI, and TTPI become more reactive than in their neutral form. These modifications could have significant consequences on their inhibitory behavior, in particular in contexts where interactions with electron-rich metal surfaces are essential.

#### 3.9.3. Frontier molecular orbitals (FMO) electron density distribution

**Fig. 13:**
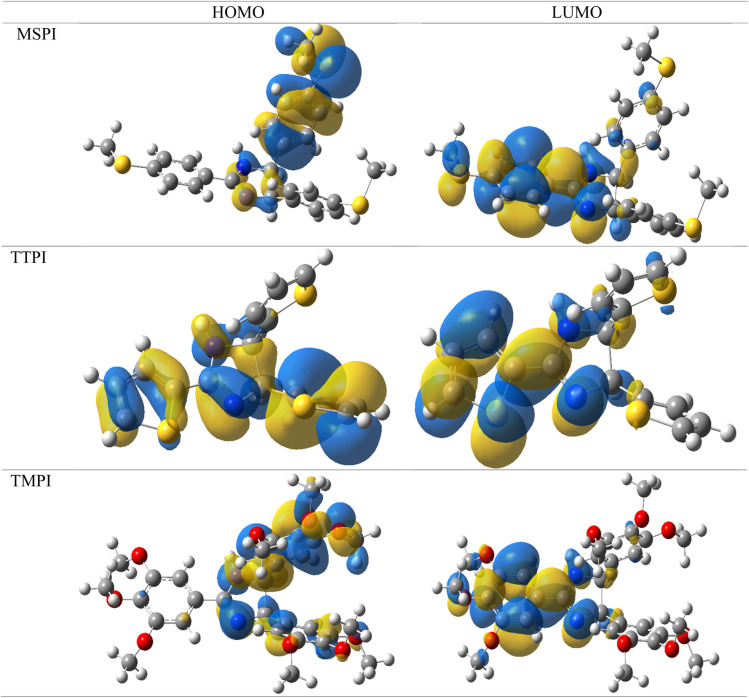
HOMO and LUMO plots of optimized MSPI, TTPI, and TMPI molecules.

The analysis of the electron density distribution of the HOMO (Highest Occupied Molecular Orbital) and LUMO (Lowest Unoccupied Molecular Orbital) frontier orbitals for the three molecules makes it possible to understand their chemical reactivity and their ability to interact with the carbon steel, considering the effects of the heteroatoms (N, O, S), the substituent groups (SCH_3_, thiophenyl and OCH_3_) and the π electrons of the phenyl rings. The electron density distribution of the HOMO and LUMO frontier molecular orbitals determined at the B3LYP/6-311G(d. p) level of theory is shown in Fig. 13.

According to Fig. 13, MSPI is distinguished by a strong localization of the HOMO on imidazole and a phenyl ring containing the SCH_3_ group, favoring strong interactions with the metal surface thanks to the non-bonding pairs of nitrogen and sulfur atoms, as well as back-donation. Its LUMO is mainly localized on the phenyl ring and the SCH_3_ group, indicating electron acceptor regions that facilitate interactions with the Fe metal. Molecule TTPI has an extensive delocalization on the thiophenyl, increasing its reactivity *via* the π electrons and the d orbitals of sulfur. Its LUMO is localized on thiophenyl and dihydroimidazoles, strengthening its ability to accept electrons and interact with the metal surface. Finally, TMPI, although less polarized, shows a localization of the HOMO on dihydroimidazoles and a phenyl ring containing the OCH_3_ groups, where the lone pairs of N atoms and the donor effect of the OCH_3_ groups facilitate interaction with the metal surface. These results explain the superior inhibitory efficiency observed experimentally, with optimal performance for sulfur-containing molecules, thanks to their electrostatic interactions and their ability to donate electrons.

#### 3.9.4. Molecular electrostatic potential (MEP) analysis

**Fig. 14:**
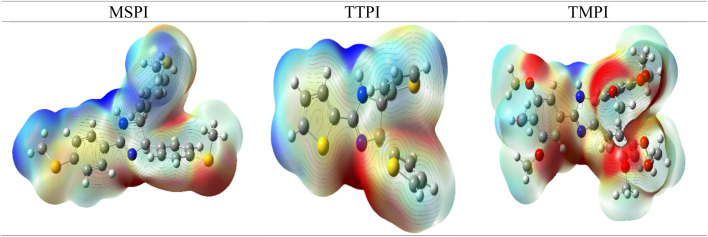
Molecular electrostatic potential surfaces plot of MSPI, TMPI, and TTPI molecules.

The MEP map (Fig. 14), determined at the DFT/B3LYP level, made it possible to identify the nucleophilic and electrophilic sites of the three (MSPI, TMPI, and TTPI) studied molecules and their interaction with the Fe(110) surface. The regions of high electron density (red zones) are located mainly on the nitrogen atoms of imidazole, the sulfur atoms of the SCH_3_ groups in molecule MSPI, and the thiophenyl in molecule TTPI, as well as on the oxygen atoms of the OCH_3_ groups in molecule TMPI. The phenyl rings also present slightly negative zones due to the delocalization of the π electrons, reinforcing the overall electron density. These electron-rich sites act as electron donors and play a key role in the interaction with the metal surface. Molecules MSPI and TTPI, due to the presence of sulfur, exhibit increased reactivity, while molecule TMPI benefits from the ability of the OCH_3_ groups to release electrons. Moreover, the electrophilic regions (blue zones) are detected around the methyl groups (CH_3_) and the H atoms, including those bound to nitrogen, indicating a low electron density. These regions, although less reactive, can interact with the metal surface through van der Waals-type interactions. Thus, the combination of heteroatoms, substituent groups, and π electrons gives MSPI, TMPI, and TTPI molecules their ability to interact effectively with the metal and to play a role in its protection against corrosion.

#### 3.9.5. Locals' active centers analysis (Fukui functions)

The reactivity of the inhibitory molecules *vis-à-vis* nucleophilic and electrophilic attacks can also be evaluated using local reactivity indices such as the dual Fukui descriptor (*f*_k_^2^), local softness (Δ*σ*_k_), and local philicity (Δ*w*_k_). These parameters make it possible to finely analyze the local reactivity of the three molecules studied, as well as the impact of chemical substitutions on their chemical properties.

The dual Fukui descriptor, the local softness (Δ*σ*_k_), and the local philicity (Δ*w*_k_) indices are calculated from the Fukui functions (*f*_k_^+^ and *f*_k_^−^), which respectively represent the tendency of a site k to undergo a nucleophilic attack (electron gain) or its sensitivity to electrophilic attacks (electron loss). Based on a Mulliken population analysis, we calculated the three descriptors per the equations:

These indices were determined based on the equations below:

13 *f*_k_^2^ = *f*_k_^+^ − *f*_k_^−^

14 Δ*σ*_k_ = *σ*_k_^+^ − *σ*_k_^−^ and Δ*ω*_k_ = *ω*_k_^+^ − *ω*_k_^−^ (*σ*_k_^+^ = *σf*_k_^+^, *σ*_k_^−^ = *σf*_k_^−^, and *ω*_k_^+^ = *ωf*_k_^+^, *ω*_k_^−^ = *ωf*_k_^−^)

**Fig. 15:**
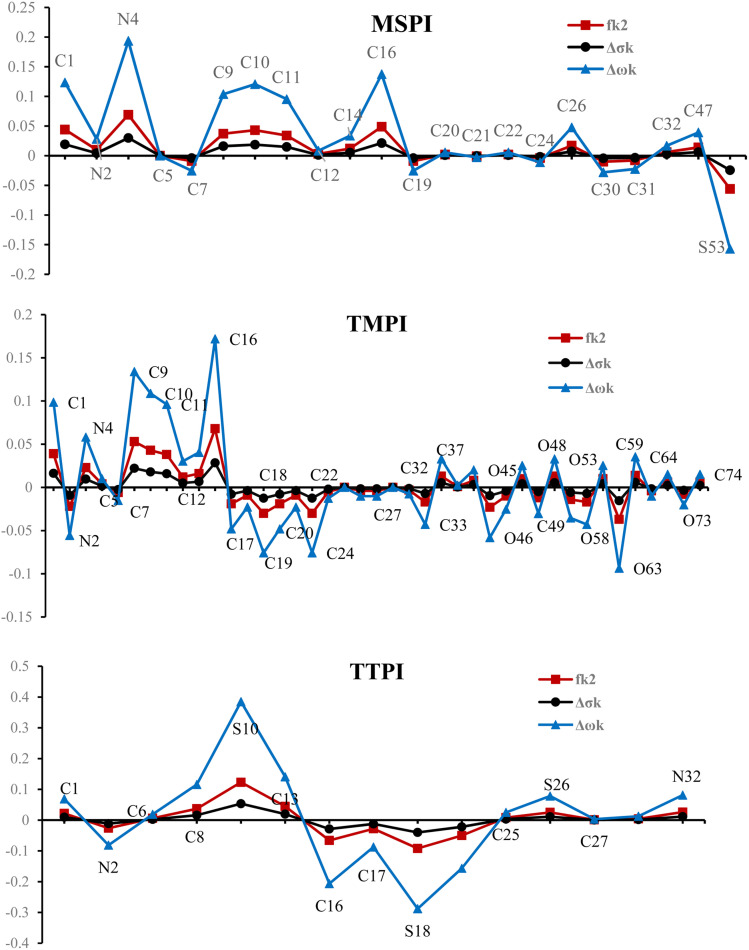
The condensed local dual descriptor, *f*_k_^2^, Δ*σ*_k_, and Δ*ω*_k_, based on Fukui Functions for MSPI, TMPI, and TTPI molecules.

The results presented graphically in Fig. 15, Table 1S, and Fig. 10S highlight the variations in the reactivity between the molecular sites. As shown in Fig. 15, in molecule MSPI, C7, C19, C30, C31, and S53 atoms have negative values of *f*_k_^2^, revealing electron-rich centers. These sites act as preferential electrophilic centers, capable of yielding electrons to the 3d vacant orbitals of the iron metal surface. This tendency is corroborated by their low values of Δ*σ*_k_ and Δ*w*_k_ indices, which confirm their willingness to release their electrons. Conversely, the atoms C1, N4, C9, C10, C11, C16 and C27, with *f*_k_^2^ > 0, are identified as nucleophilic centers. Their high values of Δ*σ*_k_ and Δ*w*_k_ emphasize their ability to accept electrons, thus participating in the formation of interaction by retro donation with the metal surface. For molecule TMPI, the centers C1, N4, C9, C10, C11, and C16 with *f*_k_^2^ > 0 behave like electrophilic sites, capable of capturing electrons from the metal, supported by their high values of Δ*σ*_k_ and Δ*w*_k_ indices. These electrophilic sites facilitate interactions with the occupied 3d orbitals of Fe. Conversely, the atoms N2, C17, C19, C20, C24, C33, O46, O49, O58, O63, and O73, characterized by a high electron density and low Δ*σ*_k_ and Δ*w*_k_ indices, act as electron donors, ideal for interacting with the metal surface. Finally, in molecule TTPI, the atoms C1, C8, S10, S26 and N36, which have *f*_k_^2^ > 0, are nucleophilic sites capable of accepting electrons, while the atoms N2, C16, C17, and S18, with their negative *f*_k_^2^ and low Δ*σ*_k_ and Δ*w*_k_ indices, act as electron-rich centers, ready to transfer them to the 3d empty orbital of the metal surface.

### 3.10. Molecular dynamics and *ab initio* DFT simulations

#### 3.10.1. MC and MD simulations

The properties of the MSPI, TMPI, and TTPI studied molecules as corrosion inhibitors were analyzed using MC and MD simulations. The objective was to examine their spontaneous interactions with the metal surface, identifying the most stable adsorption configurations. Thus, we have identified the configurations, both in the vacuum and in the corrosive environment, in their neutral or protonated forms. In addition, we determined their adsorption (*E*_ads_) and binding (*E*_bind_) energies in order to better understand their inhibitory efficacy.

**Fig. 16:**
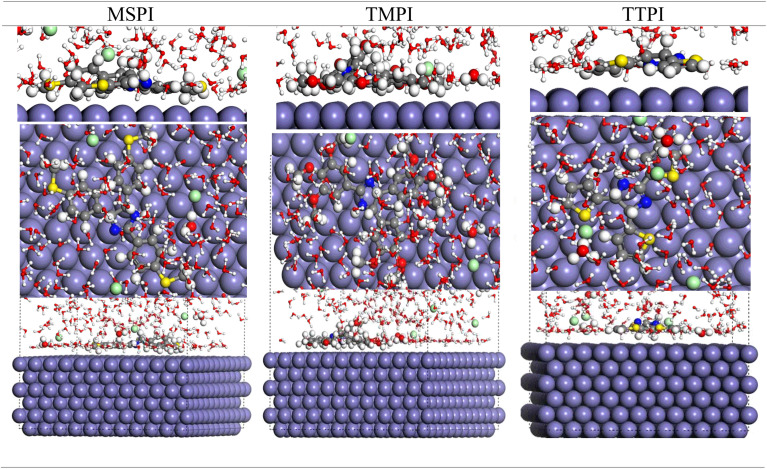
Top and side views of the most stable configurations of MSPI, TMPI, and TTPI on the Fe (110) surface in a simulated HCl medium.

The results are illustrated in Fig. 16, highlighting the optimal configurations of the three molecules adsorbed on the Fe(110) metal surface. Table 9 groups the final adsorption and binding energy values for each simulated system.

**Table 9:** Adsorption and binding energies (kcal mol^−1^) derived from MC and MD simulations

	MSPI	TMPI	TTPI
*E*_ads_ (Fe_110_ surface)	−195.916	−114.672	−123.481
*E*_ads_ (Fe_110_ surface + corrosive medium)	−4326.851	−4225.727	−4276.879
*E*_binding_	355.526	394.536	288.763

The simulations have shown that the three dihydroimidazoles studied approach the Fe(110) plane in an almost parallel orientation and adopt a finally stable and horizontal adsorption configuration on the Fe(110) surface. This flatness remains intact even in the presence of water and acid molecules, demonstrating their effectiveness in protecting this metal.

We notice that, compared with their optimized structure in the isolated state, the molecules adopt a more planar arrangement after adsorption on the metal surface, characterized by reduced dihedral angles between the outer rings and the central ring of the dihydroimidazoles. This geometry promotes optimal alignment and consequently allows them to firmly approach the metal surface and completely cover it.

The coplanar orientation of the dihydroimidazole ring with the substituted phenyl rings in MSPI and TMPI compounds (SCH_3_ and OCH_3,_ respectively), as well as with the thiophene rings in the TTPI compound, turns out to be the determining factor of the electronic behavior of these structures. Moreover, the substituents strongly influence the interactions with the iron surface, favoring stable configurations, thus helping to isolate the metal from the corrosive environment. Indeed, during the adsorption process, the substituents stabilize the adsorption configurations *via* electrostatic and steric interactions with the metal surface. They contribute to isolating the metal from the corrosive environment by effectively displacing the water molecules (H_2_O) thanks to their polar groups (OCH_3_ and sulfur-containing groups SCH_3_ and thiophenes), thus strengthening their fixation on the surface.

The adsorption energy *E*_ads_, as evaluated and reported in Table 9, quantifies the strength and stability with which the inhibitory molecules bind to the Fe(110) surface. The strongly negative values of this energy for the three molecules (−195.916, −114.672, and −123.481 Kcal mol^−1^ for MSPI, TMPI, and TTPI, respectively) testify to very strong adsorption and justify their spontaneous orientations.

In the corrosive environment, *E*_ads_ become even more pronounced (−4326.951, −4225.727, and −4276.879 kcal mol^−1^ for MSPI, TMPI, and TTPI, respectively), indicating that the molecules firmly attach to the metal by displacing the corrosive molecules of water or acid, which demonstrates the predominant affinity of the dihydroimidazoles for the carbon steel surface. This tendency is accentuated more when the dihydroimidazoles are protonated, thus reinforcing their adsorption capacity. Moreover, although all three dihydroimidazoles are effective in terms of inhibition, MSPI and TTPI display more negative interaction energies, highlighting a more marked adsorption compared to TMPI. These findings perfectly corroborate the experimental data of their inhibitory efficacy.

The molecular dynamics simulations used to determine the binding energies (*E*_binding_) corroborate the previous conclusions. The calculated values 355.526, 394.536, and 288.763 kcal mol^−1^ for MSPI, TMPI, and TTPI, respectively (Table 9), are all positive and testify to a marked binding affinity and attractive interaction between the analyzed molecules and the metal surface. These results, therefore, testify to the strength of the interaction and validate the competence of dihydroimidazoles to bind effectively to the metal surface.

#### 3.10.2. *Ab initio* DFT simulations

The global and local reactivity descriptors that we have studied in the previous sections provide valuable information on the reactivity of our organic molecules studied, but only in the isolated state. However, to better understand their behavior *vis-à-vis* the metallic iron surface and to validate the interactions mentioned above, it is interesting to resort to quantum simulations. Indeed, the DFT *ab initio* simulations make it possible to elucidate the potential interactions underlying the adsorption of the MSPI, TMPI, and TTPI inhibitors on the Fe(110) surface.

**Fig. 17:**
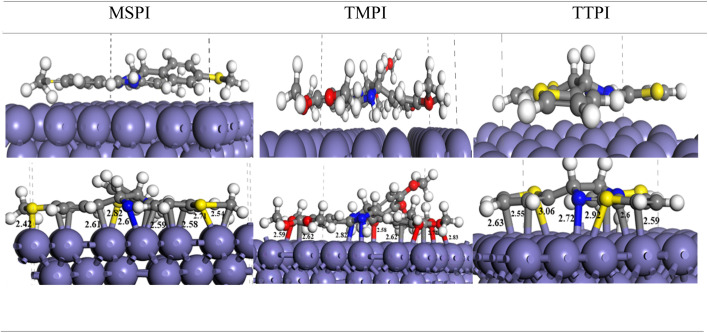
Optimized adsorption geometries of MSPI, TMPI, and TTPI molecules on the Fe (110) surface as determined by *ab initio* DFT simulations.

Fig. 17 shows the most stable adsorption geometries as well as the associated interatomic distances obtained by DFT calculation. These calculations were executed using the CASTEP software, which uses the plane wave expansion, allowing a complete exploration of the adsorption mechanism.

After optimization, the three dihydroimidazoles bind to the Fe surface in a stable parallel orientation, thus facilitating the interaction between the carbon atoms and the N, O, and S heteroatoms of the inhibitors and the Fe atoms. The molecule MSPI binds to the surface *via* its carbon, nitrogen, and sulfur atoms. The phenyl rings adsorb to the iron atoms by Fe–C bonds of about 2.6 Å, and the S atoms of the thiomethyl groups (SCH_3_) establish Fe–S bonds with lengths of 2.42 Å, 2.71 Å, and 2.82 Å, and an Fe–N bond is also observed at about 2.6 Å. The molecule TMPI interacts with the surface through several sites, involving the O atoms of the OCH_3_ groups of the two phenyl rings, as well as the C and N atoms. The Fe–O bonds vary from 2.58 to 2.83 Å, the Fe–C bonds of the phenyl rings measure approximately 2.62 Å, and nitrogen binds to the surface with 2.82 Å. As for molecule TTPI, it binds to the surface by its carbon atoms, forming Fe–C bonds of about 2.6 Å, an N atom establishes a bond of 2.82 Å, and the thiophene rings bind *via* their sulfur atoms with distances of about 3 Å.

The DFT calculations also provide estimates of the interaction energies (*E*_Int_) of the three molecules. *E*_Int_ acts as a measure of the affinity of each molecule towards the metal surface. An *E*_Int_ with more negative values means spontaneous chemical reactions, indicating superior adsorption performance. The calculation of this energy is carried out according to the following equation:

15 *E*_Int_ = *E*_TOTAL_ − (*E*_SURFACE_ − *E*_INHIBITOR_)

where *E*_TOTAL_ is the adsorption energy of the inhibitor on the surface, and *E*_SURFACE_ and *E*_INHIBITOR_, respectively, designate the energy of the surface and the energy of the isolated inhibitor. The calculated energies (Table 10) are all negative (−6.479, −4.688, and −11.741 eV for MSPI, TMPI, and TTPI, respectively), which indicates spontaneous thermodynamically favorable adsorption processes. These results show that each molecule has a strong chemical affinity for the Fe(110) surface, thanks to the engagement of several interaction centers. The SCH_3_ groups of molecules MSPI, the OCH_3_ groups of TMPI, and the thiophene rings of molecule TTPI reinforce the adsorption *via* the Fe–S and Fe–O bonds, in addition to the Fe–C bonds, thus conferring a high affinity for the Fe atoms due to their excellent electronic properties.

**Table 10:** DFT-calculated interaction energies

	Interaction energies (eV)
Fe-MSPI	−6.479
Fe-TMPI	−4.688
Fe-TTPI	−11.741

#### 3.10.3. Projected density of states (PDOS) analysis

The analysis of PDOS was carried out in order to better understand the mechanisms of adsorption and interaction between the inhibitors MSPI, TMPI, and TTPI and the Fe(110) surface. This study compares the electronic states of the molecules before and after their adsorption, thus making it possible to identify the modifications of their electronic structure. The variations observed in the PDOS provide valuable information on the electronic redistribution and the stabilization of the adsorbed molecules.

**Fig. 18:**
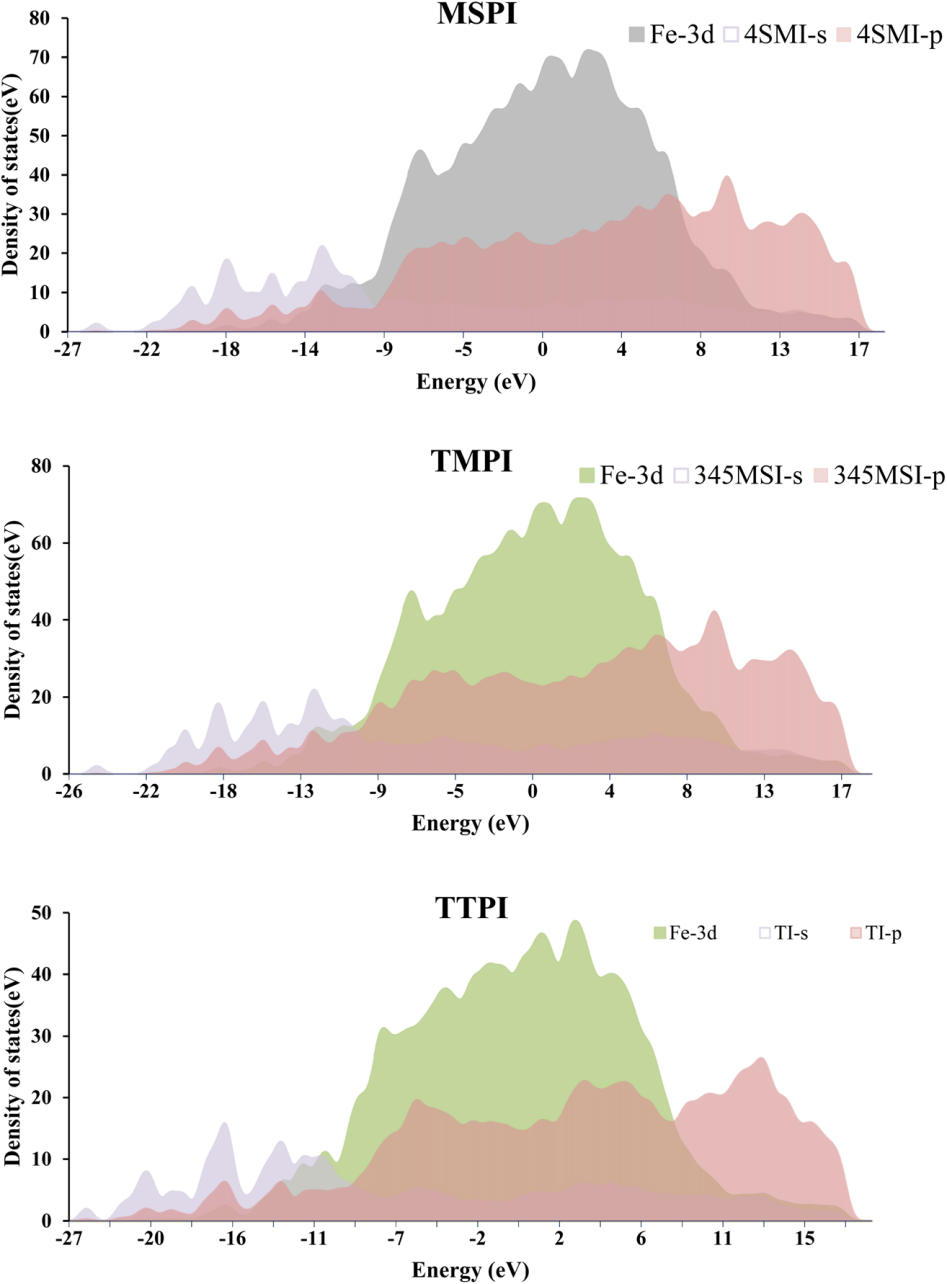
PDOS for MSPI, TMPI, and TTPI molecules on the Fe(110) surface and the Fe atoms beneath them after the adsorption.

Fig. 18 illustrates the PDOS of the 3d orbitals of Fe involved in the interaction with the molecules MSPI, TMPI, and TTPI, as well as the PDOS of the s and p orbitals of these molecules. The analysis reveals that the electronic states of the molecules evolve significantly after adsorption. A superposition of the peaks of the molecular orbitals and the iron indicates a hybridization between the d-orbitals of the Fe and the π-orbitals of the molecules, a sign of strong interaction and of the possible formation of chemical bonds. A redistribution of the electron density is also observed after adsorption, marked by a decrease in the intensity of the molecular peaks, or even their disappearance, as well as a slight shift towards lower energies, reflecting an increased stabilization of the molecules on the surface. These changes confirm an efficient adsorption, with a charge transfer to Fe. Molecules MSPI and TTPI show particularly strong interactions, as evidenced by the significant modifications of their PDOS, while molecule TMPI has similar characteristics but slightly less pronounced, reflecting equally interesting adsorption mechanisms.

## 4. Conclusion

The present investigation dissected three synthesized dihydroimidazoles as carbon steel acidizing corrosion inhibitors utilizing electrochemical, surface, and theoretical methods. The evaluation gives the following essential conclusions:

• The presence of three thiophenyl groups in TTPI, three trimethoxyphenyl groups in TMPI, and three methylthiophenyl substituents in MSPI has influenced the dihydroimidazole's effectiveness. Notably, MSPI achieved an exceptional efficacy of 99.12%, a level previously unseen for an imidazole-based inhibitor, and its efficiency remained nearly unchanged across the temperature range examined.

• Electrochemical testing revealed that the three dihydroimidazoles act as hybrid corrosion inhibitors, effectively reducing the *i*_corr_ and enhancing the *R*_p_.

• The quantum chemistry characteristics, such as the electrophilicity and nucleophilicity indices, indicate that MSPA interacts more efficiently with the steel surface, providing a strong barrier to corrosion.

• MD simulations have elucidated that the dihydroimidazole exhibits parallel orientation and adopts a finally stable and horizontal adsorption configuration on the Fe(110) surface, and their adsorption affinity is augmented when dihydroimidazoles are protonated.

## Conflicts of interest

The authors declare that they have no known competing financial interests or personal relationships that could have appeared to influence the work reported in this paper.

## Supplementary Material

RA-015-D5RA03853G-s001

## Data Availability

The data supporting the findings of this study are available within the article and its supplementary information file (SI). The supplementary information includes the full ^1^H and ^13^C NMR spectra of the synthesized dihydroimidazoles (TMPI, MSPI, and TTPI), the Fukui function descriptors, local reactivity indices, and additional computational outputs (DFT and MD simulations) that support the adsorption and inhibition analyses. Additional raw data, including original electrochemical measurements (EIS, PDP, and OCP) and high-resolution surface characterizations (SEM/EDS, AFM, XRD, and contact angle), are available from the corresponding author upon reasonable request. See DOI: https://doi.org/10.1039/d5ra03853g.
