# Supplementary material for: Effect of three substituted dihydroimidazole corrosion inhibitors on carbon steel surfaces: experimental and theoretical studies of inhibition and adsorption performance
Source: RSC Adv. 2025 Oct 21;15(47):39807–31. doi: 10.1039/d5ra03853g (PMC12538282; doi:10.1039/d5ra03853g)
Supplement: RA-015-D5RA03853G-s001 [file RA-015-D5RA03853G-s001.pdf]

Supplementary Information (SI) for RSC Advances.
This journal is © The Royal Society of Chemistry 2025

Supplementary file

Effect of three substituted dihydroimidazoles' corrosion inhibitors on carbon steel surface: Experimental and theoretical studies of inhibition and adsorption performance

A. Marzaq[1], M. El Faydy[2], Daniil R. Bazanov[3], Natalia A. Lozinskaya[3], M. Maatallah[4], G. Kaichouh[1], M. Allali[5], L. Bazzi[6], A. Dafali[2], A. Zarrouk[1,*]

[1] *Laboratory of Materials, Nanotechnology and Environment, Faculty of Sciences, Mohammed V University in Rabat. P.O. Box. 1014, Rabat, Morocco*

[2]*Laboratory of Applied Chemistry and Environment (LCAE). Faculty of Sciences. Mohammed First University. Oujda 600, Morocco*

[3]*Department of Chemistry, Lomonosov Moscow State University, 119991 Moscow, Russia*

[4]*Laboratory of Molecular Chemistry, Faculty of Sciences Semlalia, Cadi Ayyad University, PO Box 2390, Marrakech, Morocco*

[5]*Institute of Nursing Professions and Health Techniques Fez, EL Ghassani Hospital, Fez 30000, Morocco*

[6]*Laboratoire de Génie Industriel, de l'Énergétique et de l'Environnement (LGI2E), SupMTI, Rabat 10000, Morocco*

Corresponding author
Prof. Dr. Abdelkader Zarrouk
Email : azarrouk@gmail.com (AZ)
Phone: 00212665201397
Scopus Author ID: 36125763200
https://orcid.org/0009-0003-2175-7280

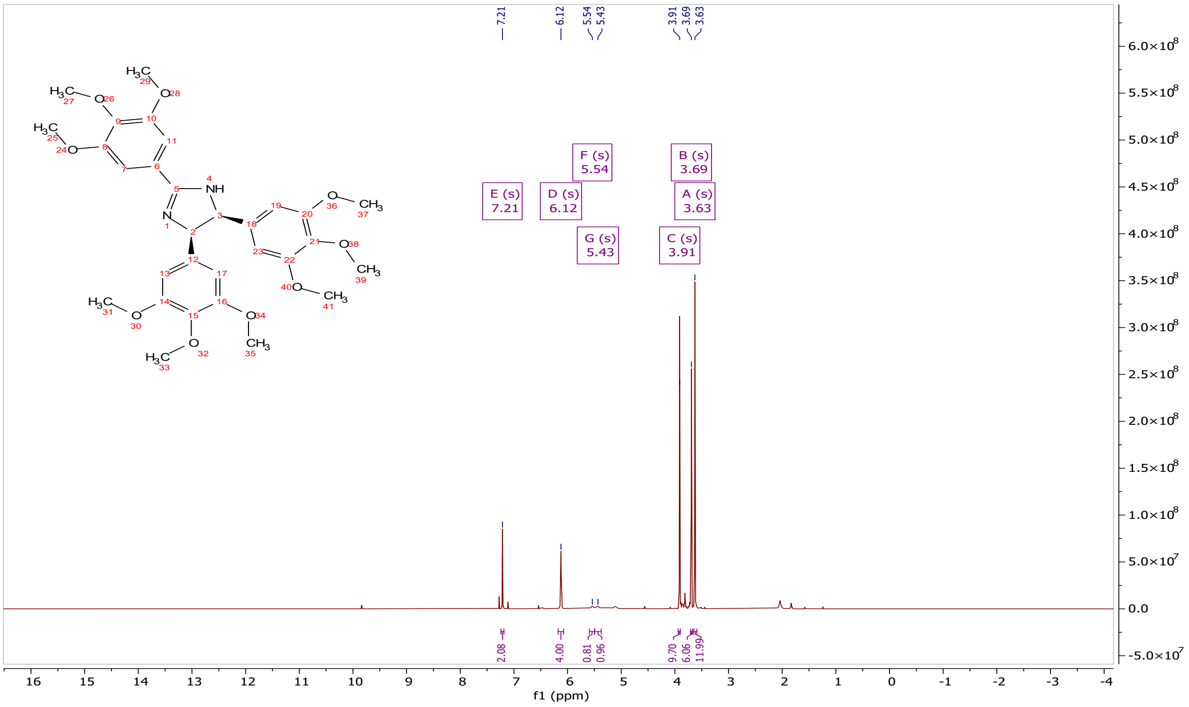

Fig. S1. ^{1}H NMR spectrum of TMPI.

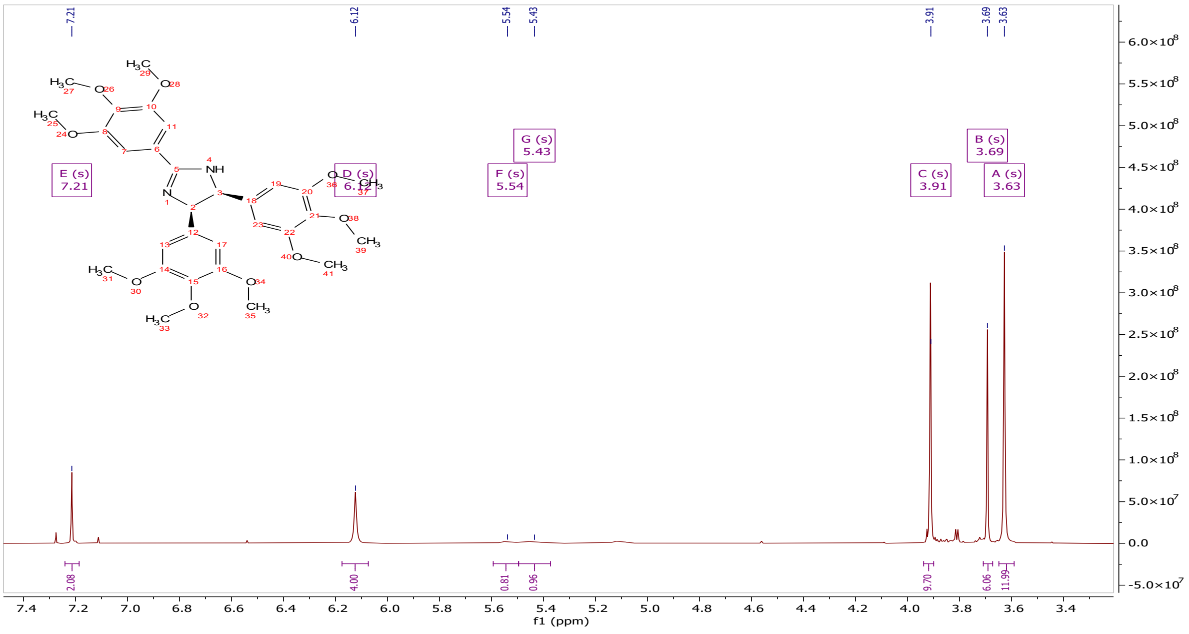

Fig. S2. ^{1}H NMR spectrum for **TMPI.**

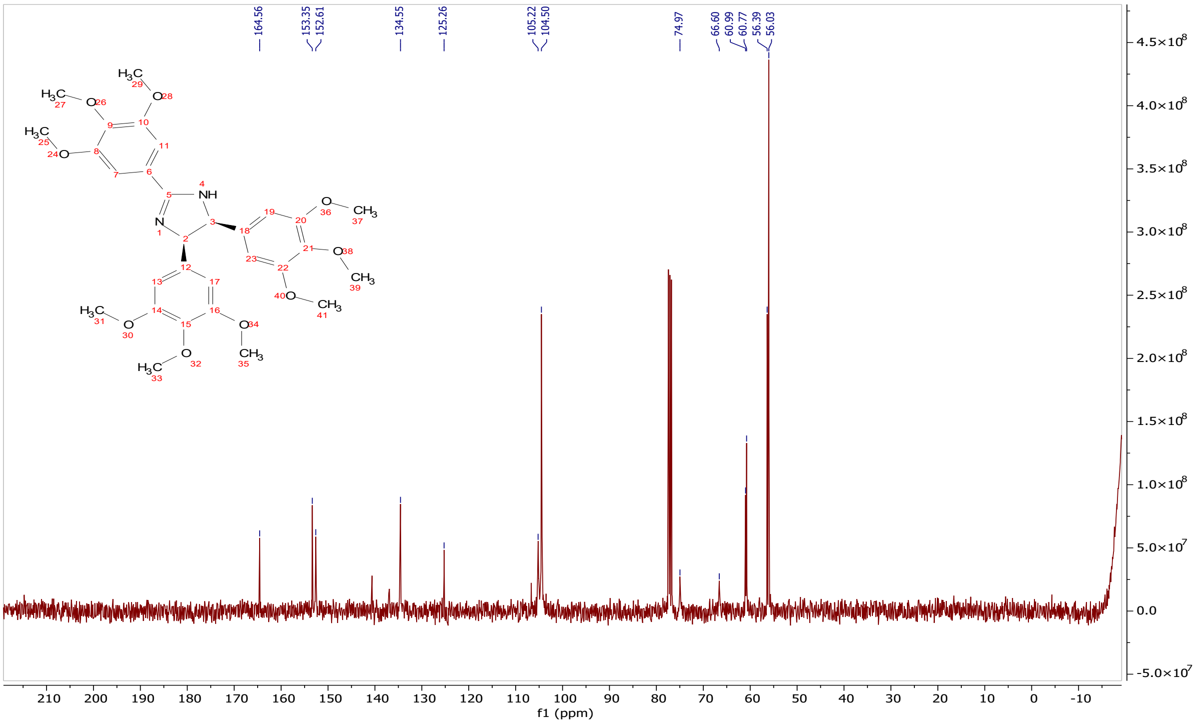

Fig. S3. ^{13}C NMR spectrum for **TMPI**.

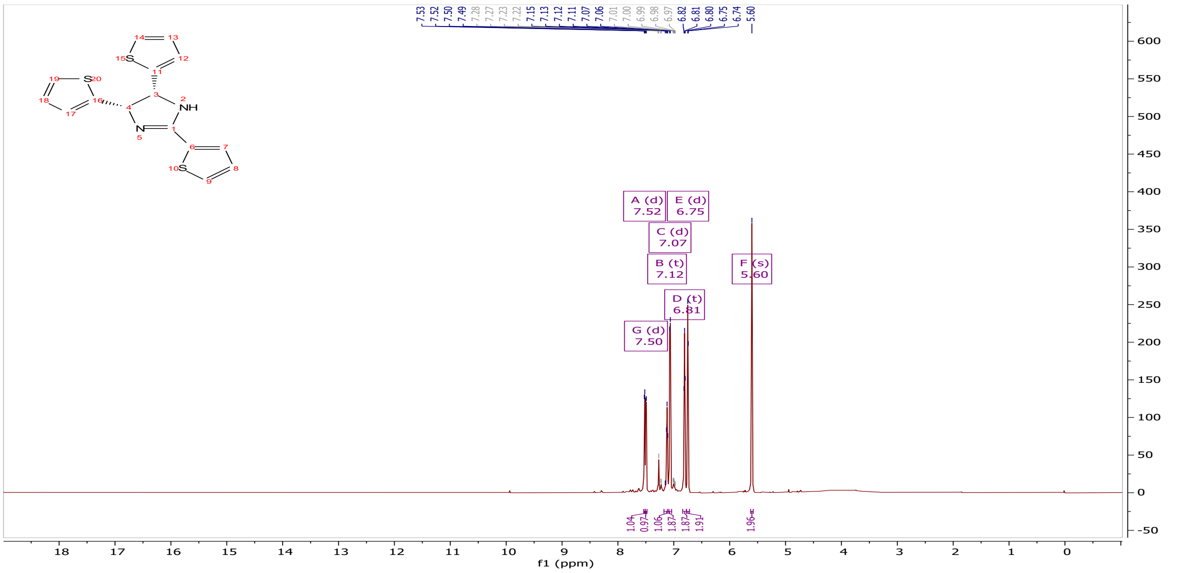

Fig. S4. ^{1}H NMR spectrum for **TTPI.**

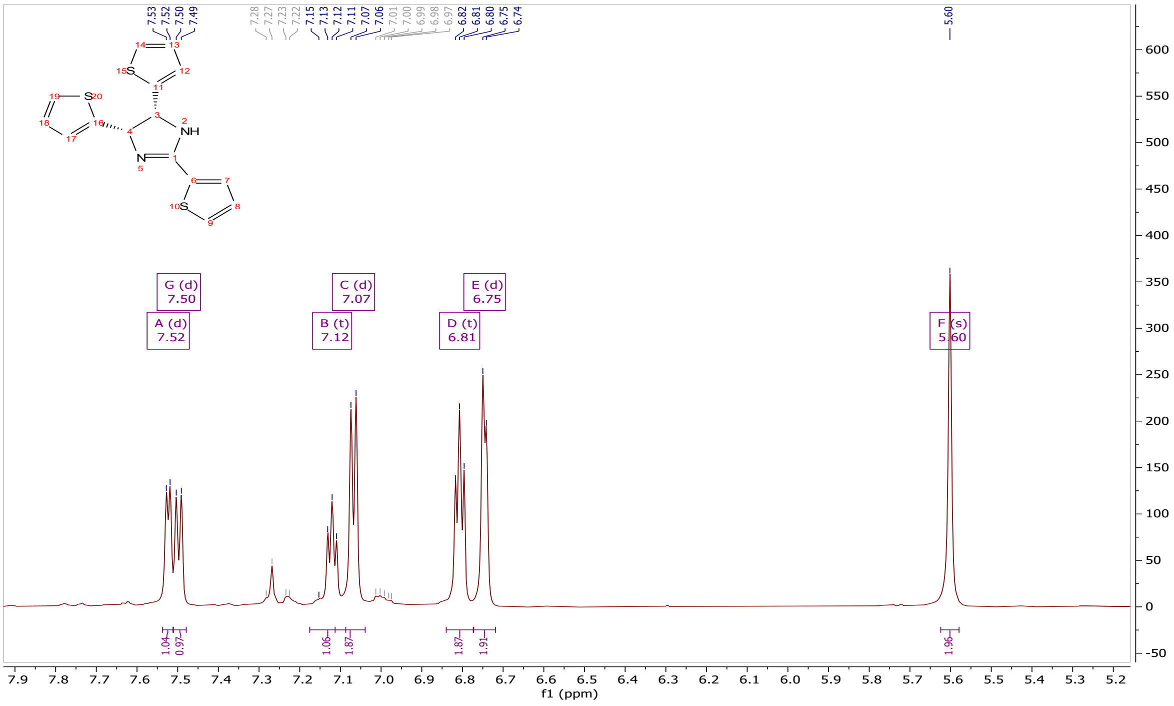

Fig. S5. ^{1}H NMR spectrum for **TTPI.**

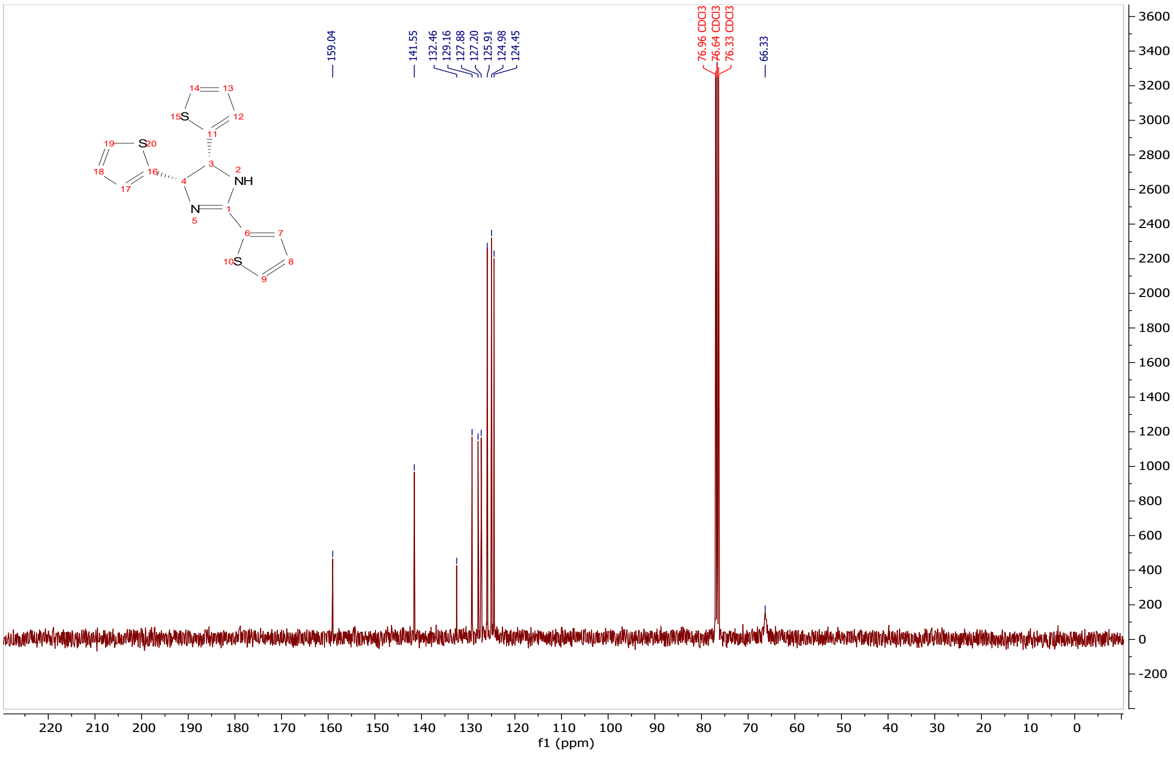

Fig. S6. ^{13}C NMR spectrum for **TTPI.**

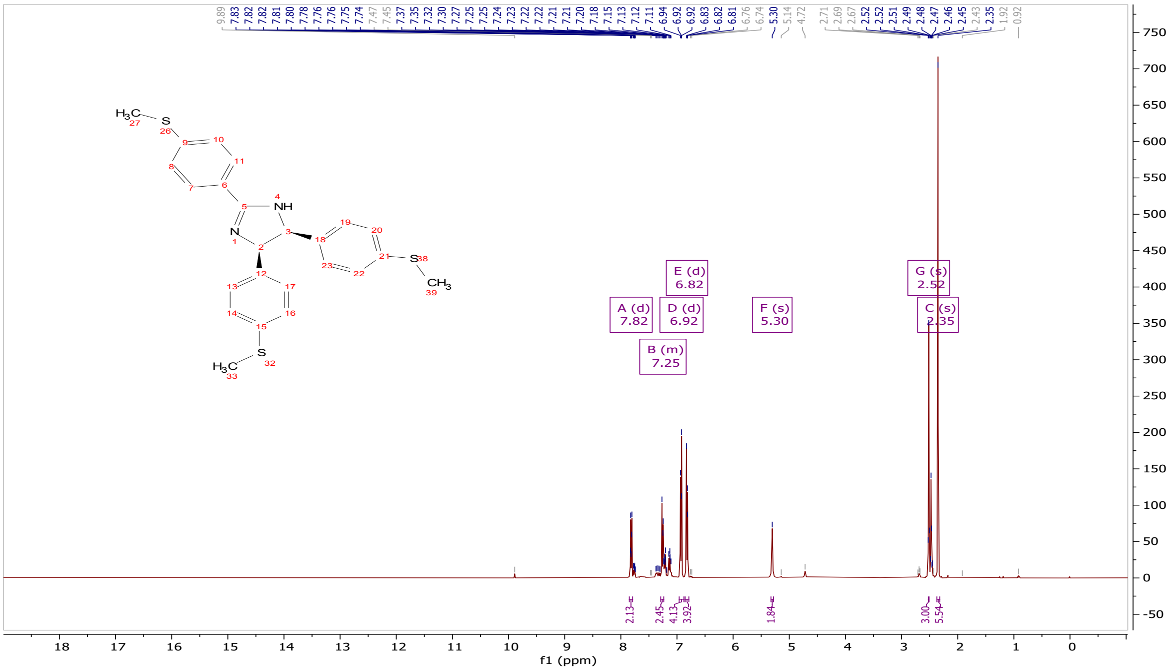

Fig. S7. ^{1}H NMR spectrum for **MSPI.**

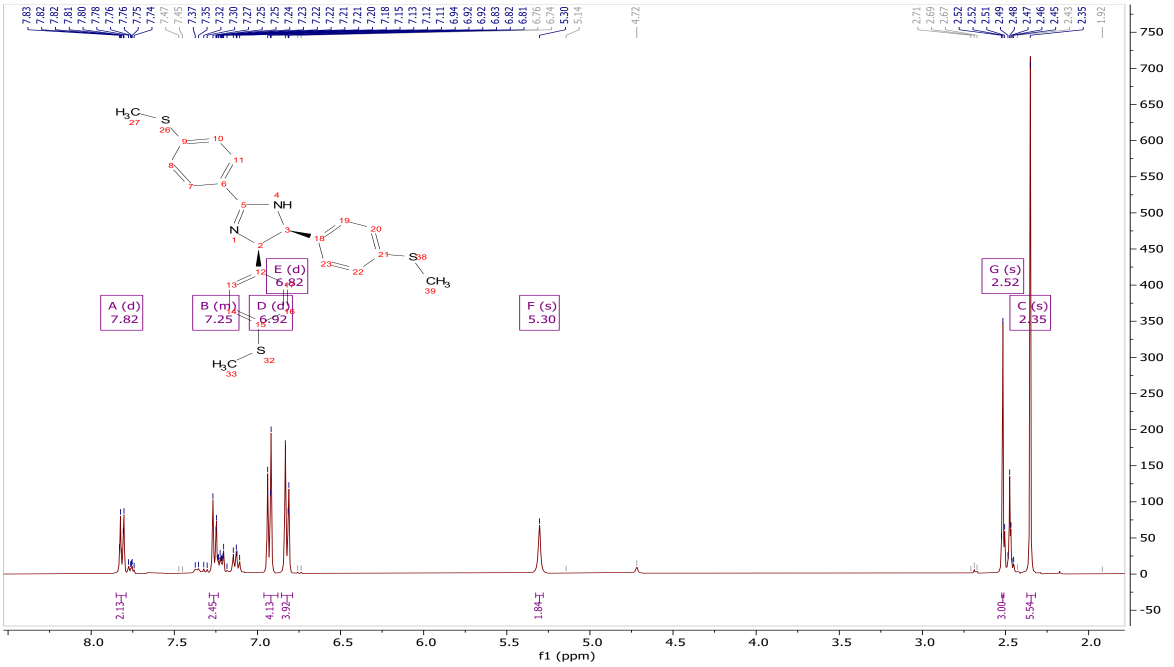

Fig. S8. ^{1}H NMR spectrum for **MSPI**.

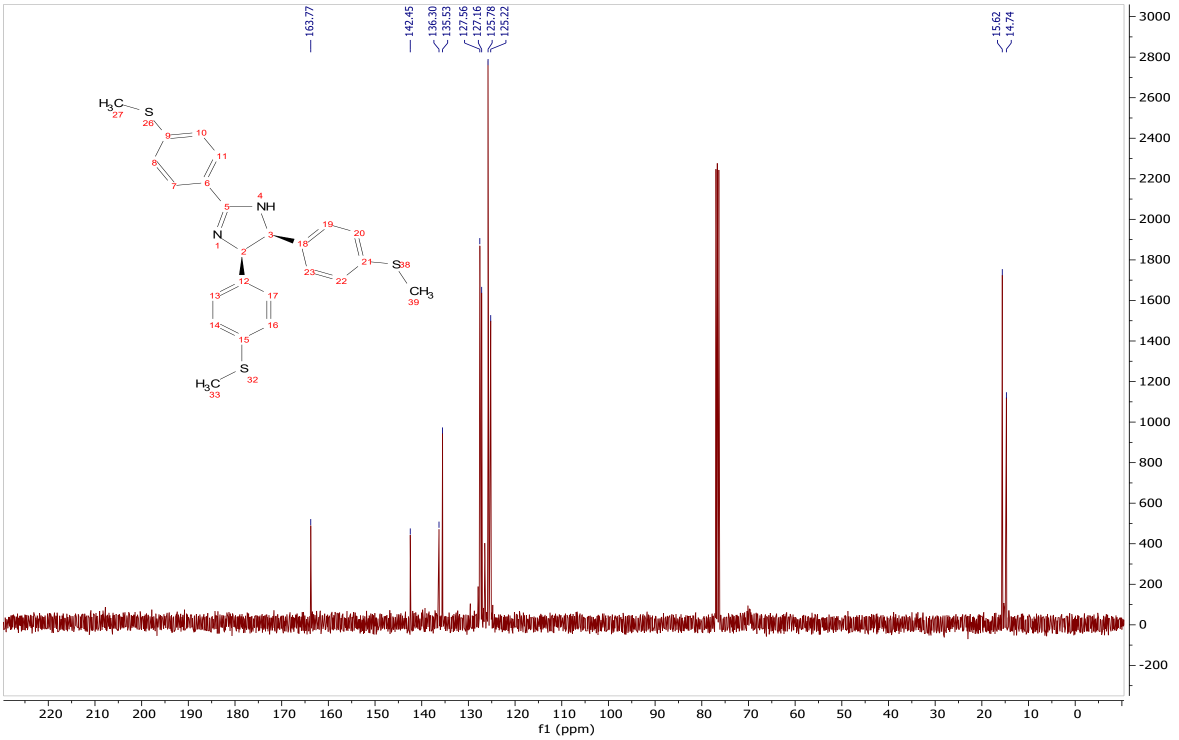

Fig. S9. ^{13}C NMR spectrum for **MSPI**.

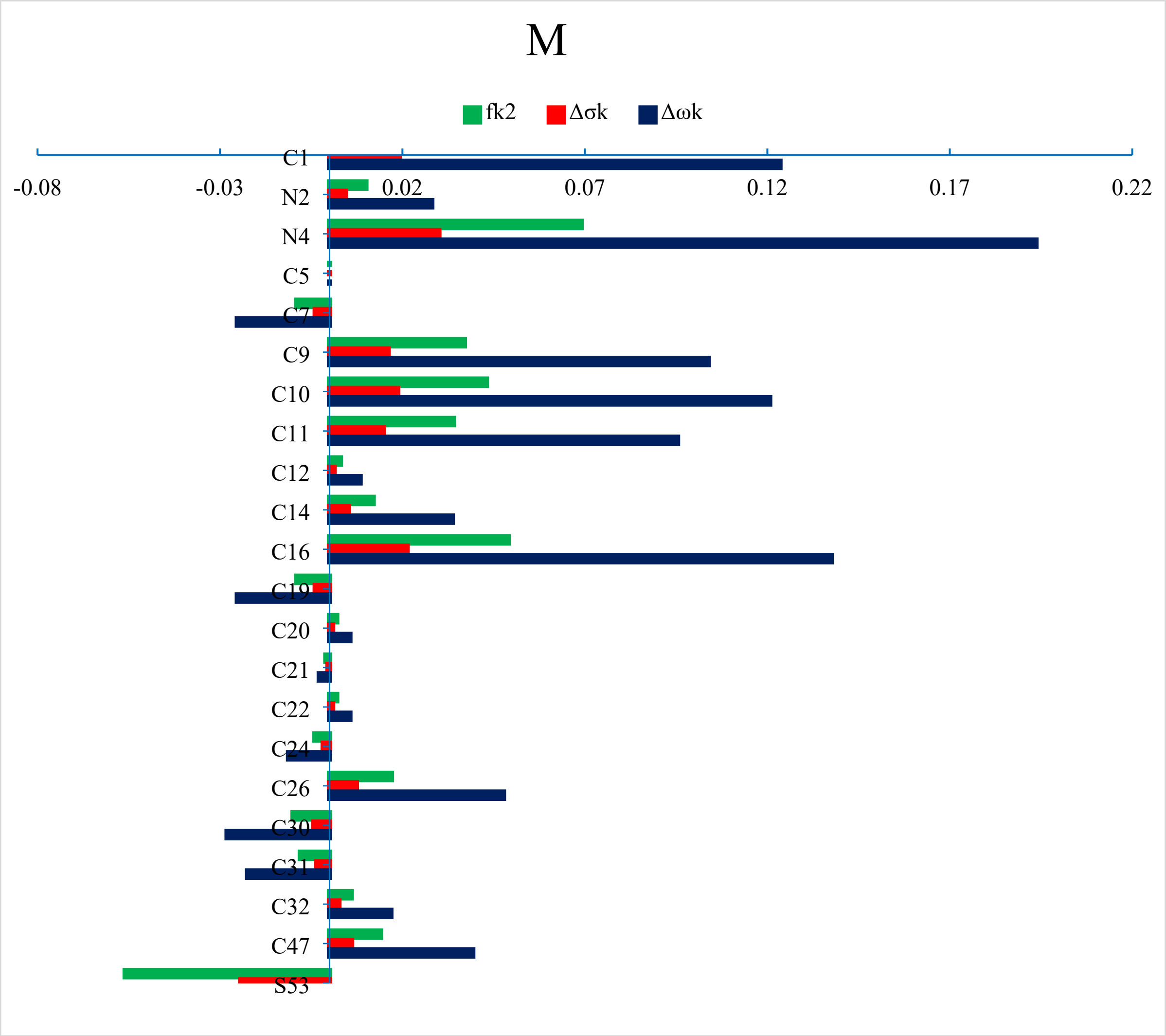

M
fk2
Δσk
Δωk
-0.08
-0.03
0.02
0.07
0.12
0.17
0.22
C1
N2
N4
C5
C7
C9
C10
C11
C12
C14
C16
C19
C20
C21
C22
C24
C26
C30
C31
C32
C47
S53

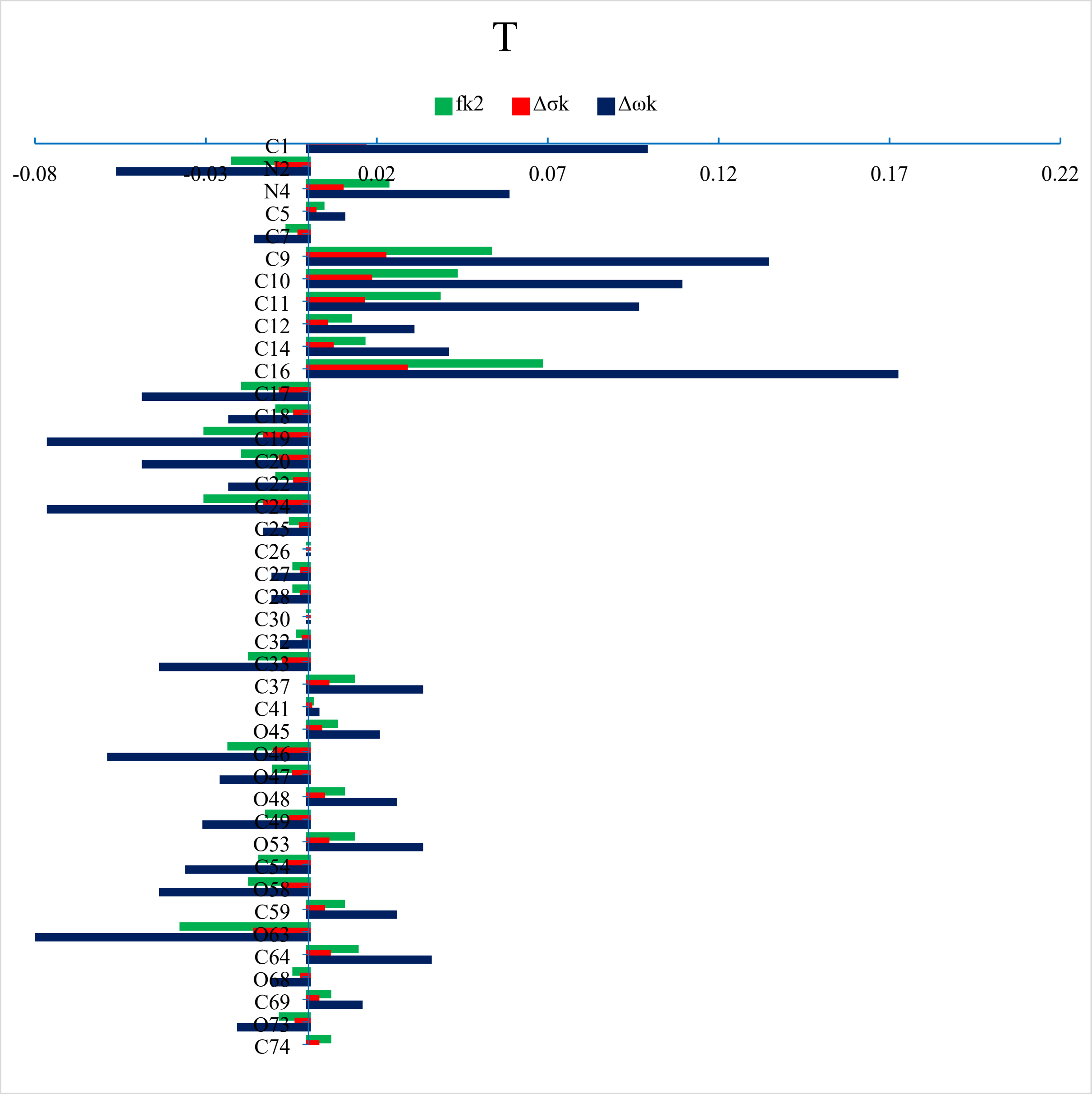
T
fk2
Δσk
Δωk
-0.08
-0.03
0.02
0.07
0.12
0.17
0.22
C1
N2
N4
C5
C7
C9
C10
C11
C12
C14
C16
C17
C18
C19
C20
C22
C24
C25
C26
C27
C28
C30
C32
C33
C37
C41
O45
O46
O47
O48
C49
O53
C54
O58
C59
O63
C64
O68
C69
O73
C74

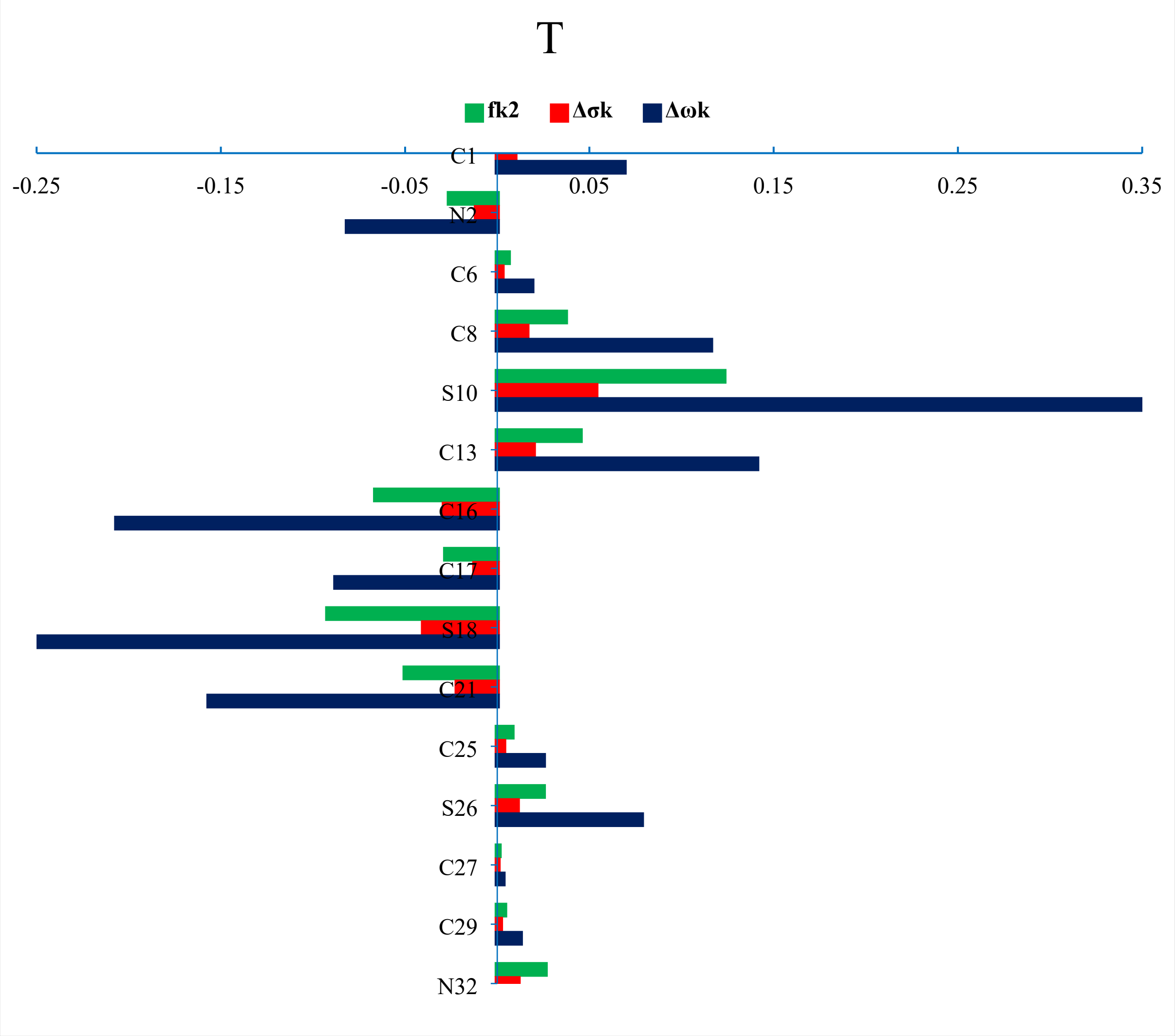

Fig. 10S. The condensed local dual descriptor, fk2, Δσk, and Δωk based on Fukui Functions for **MSPI**, **TMPI** and **TTPI** molecules.

Table 1S

Fukui indices for **MSPI**, **TMPI** and **TTPI**.

	**MSPI**		**Second degree Fukui = fk+ - fk-**	**Local softness**	**Local philicity**
Atoms	f_k^+	f_k^-	f_k^2	$\Delta\sigma_k$	$\Delta\omega_k$
**C1**	0.049	0.005	0.044	0.01913243	0.12346734
**N2**	0.019	0.009	0.01	0.00434828	0.02806076
**N4**	0.077	0.008	0.069	0.03000313	0.19361924
**C5**	-0.006	-0.006	0	0	0
**C7**	-0.015	-0.006	-0.009	-0.00391345	-0.02525468
**C9**	0.038	0.001	0.037	0.01608864	0.10382481
**C10**	0.045	0.002	0.043	0.0186976	0.12066126
**C11**	0.035	0.001	0.034	0.01478415	0.09540658
**C12**	0.011	0.008	0.003	0.00130448	0.00841823
**C14**	0.019	0.007	0.012	0.00521794	0.03367291

**C16**	0.05	0.001	0.049	0.02130657	0.13749772
**C19**	-0.002	0.007	-0.009	-0.00391345	-0.02525468
**C20**	0.003	0.001	0.002	0.00086966	0.00561215
**C21**	0.002	0.003	-0.001	-0.00043483	-0.00280608
**C22**	0.008	0.006	0.002	0.00086966	0.00561215
**C24**	0.006	0.01	-0.004	-0.00173931	-0.0112243
**C26**	0.006	-0.011	0.017	0.00739208	0.04770329
**C30**	-0.003	0.007	-0.01	-0.00434828	-0.02806076
**C31**	-0.004	0.004	-0.008	-0.00347862	-0.02244861
**C32**	0.007	0.001	0.006	0.00260897	0.01683646
**C47**	-0.004	-0.018	0.014	0.00608759	0.03928506
**S53**	0.041	0.097	-0.056	-0.02435037	-0.15714025

	**TMPI**		**Second degree Fukui = fk+ - fk-**	**Local softness**	**Local philicity**
Atoms	f_k^+	f_k^-	f_k^2	$\Delta\sigma_k$	$\Delta\omega_k$
**C1**	0.049	0.01	0.039	0.01628605	0.09858263
**N2**	0.021	0.043	-0.022	-0.009187	-0.05561071
**N4**	0.081	0.058	0.023	0.00960459	0.05813847
**C5**	-0.007	-0.011	0.004	0.00167036	0.01011104
**C7**	-0.016	-0.01	-0.006	-0.00250555	-0.01516656
**C9**	0.046	-0.007	0.053	0.02213232	0.13397126
**C10**	0.044	0.001	0.043	0.01795641	0.10869366
**C11**	0.041	0.003	0.038	0.01586846	0.09605487
**C12**	0.025	0.013	0.012	0.00501109	0.03033312
**C14**	0.028	0.012	0.016	0.00668145	0.04044415
**C16**	0.089	0.021	0.068	0.02839618	0.17188766
**C17**	-0.001	0.018	-0.019	-0.00793423	-0.04802743
**C18**	0.004	0.013	-0.009	-0.00375832	-0.02274984
**C19**	-0.002	0.028	-0.03	-0.01252773	-0.07583279
**C20**	0.009	0.028	-0.019	-0.00793423	-0.04802743
**C22**	0.009	0.018	-0.009	-0.00375832	-0.02274984
**C24**	0.016	0.046	-0.03	-0.01252773	-0.07583279
**C25**	-0.006	-0.001	-0.005	-0.00208795	-0.0126388
**C26**	0	0	0	0	0
**C27**	-0.003	0.001	-0.004	-0.00167036	-0.01011104
**C28**	0.01	0.014	-0.004	-0.00167036	-0.01011104
**C30**	0.009	0.009	0	0	0
**C32**	0.014	0.017	-0.003	-0.00125277	-0.00758328
**C33**	-0.026	-0.009	-0.017	-0.00709905	-0.04297191
**C37**	-0.009	-0.022	0.013	0.00542868	0.03286088
**C41**	-0.006	-0.007	0.001	0.00041759	0.00252776
**O45**	0.019	0.011	0.008	0.00334073	0.02022208
**O46**	0.006	0.029	-0.023	-0.00960459	-0.05813847

**O47**	0.007	0.017	-0.01	-0.00417591	-0.0252776
**O48**	0.02	0.01	0.01	0.00417591	0.0252776
**C49**	-0.023	-0.011	-0.012	-0.00501109	-0.03033312
**O53**	0.018	0.005	0.013	0.00542868	0.03286088
**C54**	-0.023	-0.009	-0.014	-0.00584627	-0.03538864
**O58**	0.002	0.019	-0.017	-0.00709905	-0.04297191
**C59**	-0.006	-0.016	0.01	0.00417591	0.0252776
**O63**	0.01	0.047	-0.037	-0.01545086	-0.09352711
**C64**	-0.007	-0.021	0.014	0.00584627	0.03538864
**O68**	0.001	0.005	-0.004	-0.00167036	-0.01011104
**C69**	-0.007	-0.013	0.006	0.00250555	0.01516656
**O73**	0.005	0.013	-0.008	-0.00334073	-0.02022208
**C74**	-0.009	-0.015	0.006	0.00250555	0.01516656

	**TTPI**		**Second degree Fukui= fk+ - fk-**	**Local softness**	**Local philicity**
Atoms	f_k^+	f_k^-	f_k^2	$\Delta\sigma_k$	$\Delta\omega_k$
**C1**	0.051	0.029	0.022	0.00957074	0.068838
**N2**	0.02	0.046	-0.026	-0.01131088	-0.081354
**C6**	-0.016	-0.022	0.006	0.0026102	0.018774
**C8**	0.033	-0.004	0.037	0.01609625	0.115773
**S10**	0.17	0.047	0.123	0.05350916	0.38486699
**C13**	0.074	0.029	0.045	0.01957652	0.140805
**C16**	-0.019	0.047	-0.066	-0.02871223	-0.206514
**C17**	0.007	0.035	-0.028	-0.01218095	-0.087612
**S18**	0.001	0.093	-0.092	-0.04002311	-0.28786799
**C21**	0.012	0.062	-0.05	-0.02175169	-0.15645
**C25**	0.004	-0.004	0.008	0.00348027	0.025032
**S26**	0.047	0.022	0.025	0.01087584	0.078225
**C27**	0.005	0.004	0.001	0.00043503	0.003129
**C29**	0.017	0.013	0.004	0.00174014	0.012516
**N32**	0.082	0.056	0.026	0.01131088	0.081354
